# Murine AML12 hepatocytes allow *Salmonella* Typhimurium T3SS1-independent invasion and intracellular fate

**DOI:** 10.1038/s41598-021-02054-z

**Published:** 2021-11-23

**Authors:** S. Holbert, E. Barilleau, S. M. Roche, J. Trotereau, S. Georgeault, J. Burlaud-Gaillard, A. Wiedemann, S. Méresse, I. Virlogeux-Payant, P. Velge

**Affiliations:** 1 ISP, INRAE, Université de Tours, Nouzilly, France; 2 Plateforme IBiSA de Microscopie Electronique, Université de Tours et CHRU de Tours, Tours, France; 3 CNRS, INSERM, CIML, Aix Marseille Univ, Marseille, France

**Keywords:** Cell biology, Microbiology

## Abstract

Numerous studies have demonstrated the key role of the *Salmonella* Pathogenicity Island 1-encoded type III secretion system (T3SS1) apparatus as well as its associated effectors in the invasion and intracellular fate of *Salmonella* in the host cell. Several T3SS1 effectors work together to control cytoskeleton networks and induce massive membrane ruffles, allowing pathogen internalization. *Salmonella* resides in a vacuole whose maturation requires that the activity of T3SS1 subverts early stages of cell signaling. Recently, we identified five cell lines in which *Salmonella* Typhimurium enters without using its three known invasion factors: T3SS1, Rck and PagN. The present study investigated the intracellular fate of *Salmonella* Typhimurium in one of these models, the murine hepatocyte cell line AML12. We demonstrated that both wild-type *Salmonella* and T3SS1-invalidated *Salmonella* followed a common pathway leading to the formation of a *Salmonella* containing vacuole (SCV) without classical recruitment of Rho-GTPases. Maturation of the SCV continued through an acidified phase that led to *Salmonella* multiplication as well as the formation of a tubular network resembling *Salmonella* induced filaments (SIF). The fact that in the murine AML12 hepatocyte, the T3SS1 mutant induced an intracellular fate resembling to the wild-type strain highlights the fact that *Salmonella* Typhimurium invasion and intracellular survival can be completely independent of T3SS1.

## Introduction

*Salmonella enterica* subsp. *enterica* serovar Typhimurium (*S.* Typhimurium) is the causative agent responsible for the second most deadly foodborne infection known as salmonellosis^1^. In humans, *S.* Typhimurium induces gastroenteritis characterized by fever, acute intestinal inflammation and diarrhea within 24 h of infection. In animals, *S.* Typhimurium is commonly isolated from healthy birds and mammals. However, it can also induce a systemic typhoid-like disease or gastroenteritis in mice or calves, respectively^2^. This facultative intracellular pathogen needs to cross intestinal barriers to be able to infect its hosts. Therefore, it must adapt to invade, survive and replicate within phagocytic and non-phagocytic cells^3^.

*Salmonella* has two *Salmonella* Pathogenicity Islands (SPI) that encode type III secretion systems (T3SSs). They are T3SS1 and T3SS2, encoded by SPI1 and SPI2, respectively. The T3SSs consist of a needle apparatus that injects effector proteins into host target cells. They are the key factors involved in the interaction of bacteria with host cells. Previously, only T3SS1-dependent invasion of non-phagocytic cells had been described. However, several studies agreed that *Salmonella* with a non-functional T3SS1 was able to induce pathologies in humans or experimentally infected animals^4,5^. Moreover, in vitro studies have shown that *Salmonella* invalidated for T3SS1 maintained its invasive ability in fibroblastic cells^6^, and required kinases that was not necessary in T3SS1-dependent invasion cell model^6^. In light of these results, two invasins, Rck and PagN, have been identified in the invasion process^7,8^. We recently published that *Salmonella* invalidated for the three known invasion factors (T3SS1 apparatus, Rck and PagN) remains able to invade several non-phagocytic cell models as effectively as wild-type *Salmonella*^9^. The main difference in the entry step between T3SS1-dependent and T3SS1-independent invasion is that T3SS1 effectors induce cell invasion by means of a trigger mechanism, while invasins mediate invasion using a zipper mechanism characterized by the interaction between a bacterial outer membrane protein and a host receptor^10^. The intracellular behavior of *Salmonella* in a vacuole called SCV (*Salmonella* containing vacuole) after a T3SS1-dependent entry has been well documented. Several effectors translocated by T3SS1 or T3SS2 have been shown to interact directly with host cell partners^11^ and are involved in SCV formation and maturation. Among the T3SS1 effectors, two play key roles. The first, SipA, polymerizes actin and participates in triggering large membrane ruffles, leading to *Salmonella* engulfment^12^. By mimicry of proteins implicated in vesicular transport, it then promotes fusion with early endosomes^13^. The second, SopB, not only plays a role in actin remodeling, but also induces Rab5 recruitment, required for optimal biogenesis of SCV through its phosphatase activity^14^. On the other hand, several T3SS2 effectors are implicated especially in SCV maturation, following well-described kinetics dependent on transient SCV interaction with late endosomal proteins such as Rab7. Among these effectors, SopD2 interacts directly with Rab7 and inhibits vesicular transport, thus favoring vacuole maturation^15,16^. This is characterized by the recruitment of late endosomal proteins, such as the lysosomal-associated membrane protein 1 (LAMP1)^17^. SifA is then required for the formation of *Salmonella*-induced filaments (SIFs) and hence SCV maturation^18^. Moreover, persistent expression of T3SS1 effectors during late vacuole maturation steps highlights the complexity of SCV biogenesis as well as the close communication between *Salmonella* and its host, allowing multiplication inside the vacuole while maintaining SCV integrity^19^. However, it has been shown that *Salmonella* is able to escape from its early vacuole into the cytosol in several cell types as well as in vivo^20,21^. The host cytosol environment is favorable for bacterial growth and hyper-replication leading to epithelial cell lysis and bacterial release^21,22^. In addition to its role in *Salmonella* invasion and in SCV maturation, T3SS1 is also required for vacuolar escape and replication in the cytosol of epithelial cells^23^. Nevertheless, SCV maturation and cytosolic escape have only been described in cells where *Salmonella* enters in a T3SS1-dependent manner. Nothing is known about *Salmonella* behavior after T3SS1-independent cell invasion.

Here, our goal was to depict *S.* Typhimurium fate inside host cells in a T3SS1-independent invasion cell model and to determine if the T3SS1 remained indispensable in survival of *S.* Typhimurium and maturation of SCV as observed in T3SS1-dependent invasion models. We showed that a wild-type strain of *S.* Typhimurium invaded the murine hepatocyte cell line AML12 through a T3SS1-independent process and retained its ability to multiply in a mature vacuole, it had a functional T3SS1 or not.

## Results

### A *Salmonella* Typhimurium Δ*invA* mutant enters, survives and multiplies in the same manner as the wild-type strain in murine hepatocytes

**Figure 1:**
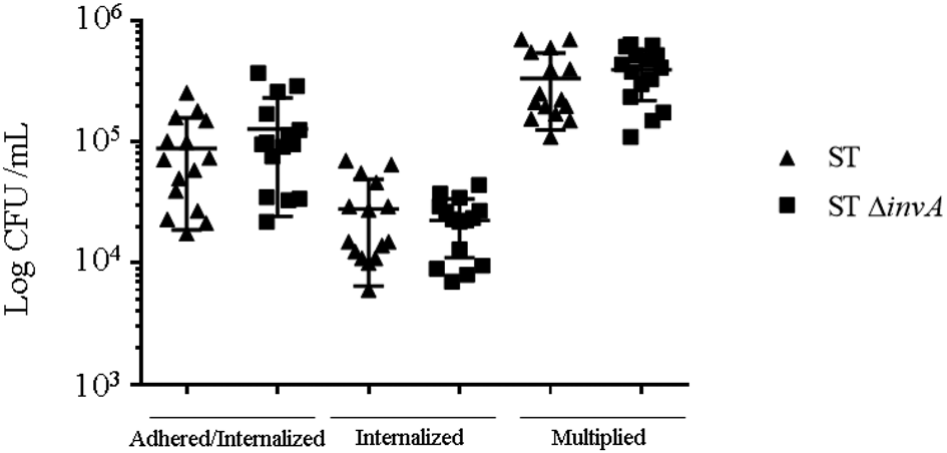
*S.* Typhimurium 14028 inactivated for T3SS1 activity by *invA* mutation remained as invasive as the wild-type strain in the AML12 cell model. In repeated gentamicin protection assays, adhered, internalized and multiplied ST (black triangles) or ST Δ*invA* (black squares) were monitored on AML12 cells. Cells were exposed to each strain (MOI = 50) for 60 min (adhesion/invasion) followed by the addition of gentamicin (100 μg/mL) for 60 min (Invasion), followed by a further 15 h with gentamicin (10 µg/mL) (multiplication). The results correspond to the mean ± standard deviation of at least four independent experiments performed in quadruplicate and expressed in log CFU/well. Results were compared using a Mann–Whitney test. No significant differences were observed between the two strains.

We have recently described that *S.* Typhimurium 14028, devoid of the three known invasion factors (i.e. T3SS1, Rck and PagN), remained capable of invading several non-phagocytic cell models at a similar rate to the wild-type strain, including murine AML12 hepatocytes^9^. In order to evaluate the ability of *Salmonella* to survive and multiply in the AML12 cell line, we first confirmed the similar invasiveness of a non-functional T3SS1 *S.* Typhimurium 14028 mutant (ST Δ*invA*) and its *S.* Typhimurium 14028 wild-type parent (ST). ST and ST Δ*invA* were grown overnight, a condition known to weakly induce the expression of Rck^24^ and PagN^25^ (Supplementary Fig. 1). HeLa cells were used as a T3SS1-dependent reference cell model. Adhesion and invasion of HeLa or AML12 cells were evaluated after 60 min of bacterial-cell contact. The level of total bacteria, corresponding to adhered and invaded bacteria, was almost equivalent in both HeLa and AML12 cells. No differences between the wild-type and the Δ*invA* mutant were observed (Fig. 1 and Supplementary Fig. 2). As predicted, HeLa cells showed an elevated level of T3SS1-dependent invasion. Indeed, the Δ*invA* mutant invaded these cells 50 times less than the wild-type strain (Supplementary Fig. 2). In contrast, no significant difference was observed between the two strains after infection of AML12 cells (Fig. 1). To avoid investigating an artifactual mechanism due to exacerbated AML12 cells’ permissiveness, we quantified the invasion and multiplication ability of two non-invasive *E. coli* strains: MC1061 and HB101. As expected, MC1061 and HB101 entered AML12 cells respectively 52.03 (*p* < 0.0001) and 205.4 (*p* < 0.0001) times less than ST and did not multiply (Supplementary Fig. 2). To evaluate the intracellular multiplication of *Salmonella*, we next quantified the number of intracellular *Salmonella* 16 h pi in AML12 and HeLa cells. We observed an increase of *Salmonella* in AML12 cells of more than 10 times with no significant difference (*p* = 0.6514) between ST and ST Δ*invA* strains (Fig. 1). In contrast, a significant difference between the multiplication rates of ST and ST Δ*invA* in HeLa cells was obtained: an increase of 18 times for ST compared to an increase of only 6 times for ST Δ*invA* (*p* = 0.0039). No *E coli* multiplication was observed in both cell lines. Altogether, these results demonstrate that a Δ*invA* mutant of *Salmonella* Typhimurium invaded, survived and multiplied equally well as the wild-type strain in the non-phagocytic AML12 cells.

### In AML12 cells, the wild-type strain of *Salmonella* Typhimurium 14028 rarely translocates T3SS1 effectors and preferentially invades cells using a zipper mechanism

**Figure 2:**
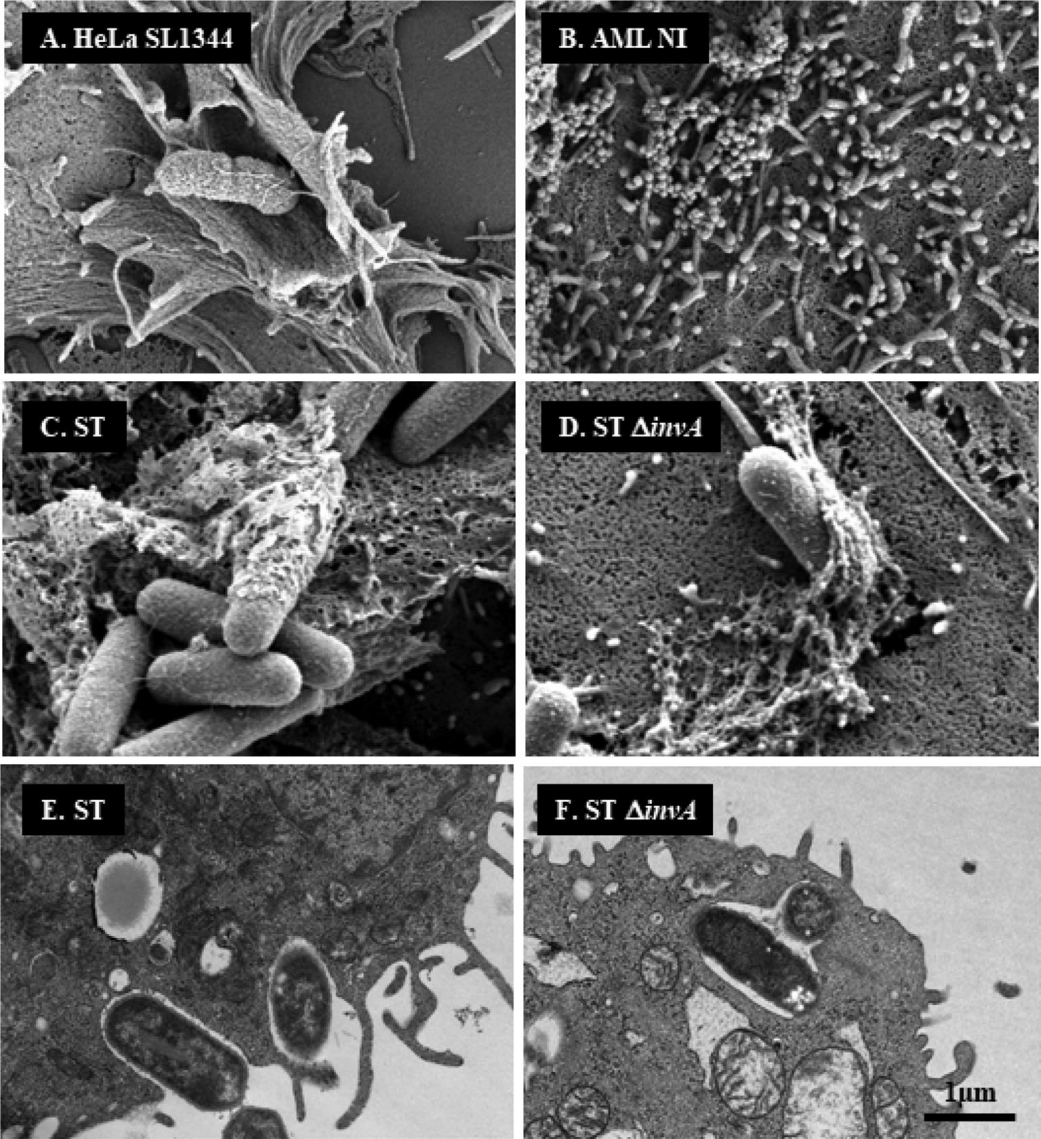
Weak cell membrane rearrangements observed by electron microscopy during invasion illustrate a zipper mechanism for both ST and ST Δ*invA* strains in AML12 cells. (**A**) HeLa cells infected for 30 min with *Salmonella* Typhimurium (SL1344) as a trigger reference model. (**B**) AML12 cells not infected, or (**C**,**E**) infected with ST or (**D**,**F**) infected with ST Δ*invA* for 60 min. (**A**–**D**) Infected HeLa cells and non-infected or infected AML12 cells cultured on glass coverslips were used to evaluate membrane ruffling using scanning electron microscopy. (**E**,**F**) Infected AML12 cells were trypsinized and used for transmission electron microscopy observation of ultra-thin sections of embedded cells in resin after fixation and impregnation. Scale bars 1 µm.

**Figure 3:**
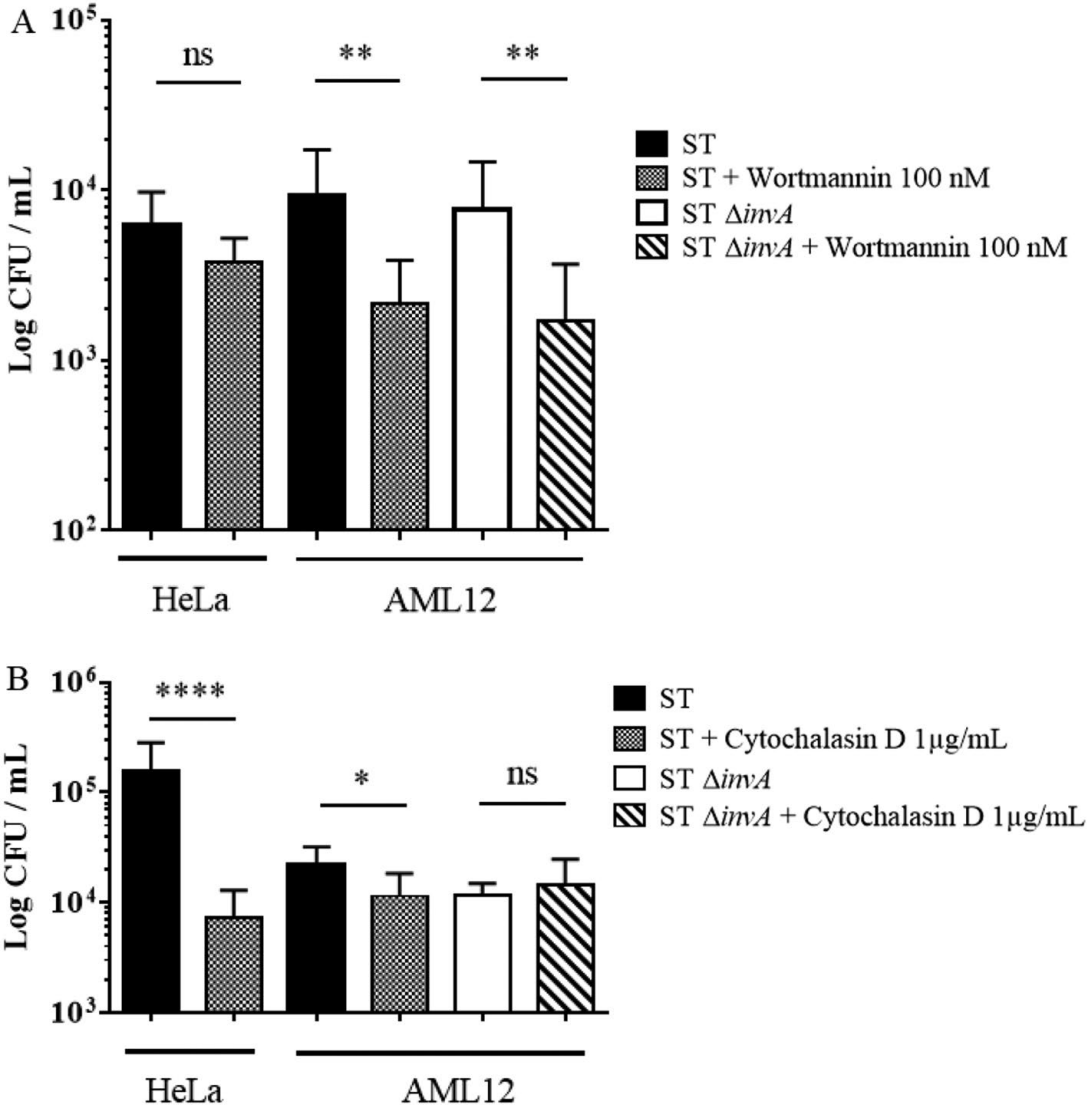
*Salmonella* Typhimurium entry into AML12 cells was dependent on the PI3 kinase pathway and required weak actin cytoskeletal rearrangements. HeLa cells and AML12 cells were exposed to (**A**) wortmannin (100 nM) (Sigma, St-Louis, MO), a PI3 kinase inhibitor, for 30 min or to (**B**) cytochalasin D (1 µg/mL) for 30 min, followed by an infection and gentamicin protection assay with ST and ST Δ*invA*. Data are the means and standard deviations from three independent experiments. Statistical analyses using a Mann Whitney test were performed. Significances were (****) *p* < 0.0001; (**) *p* < 0.01; (*) *p* < 0.05.

**Figure 4:**
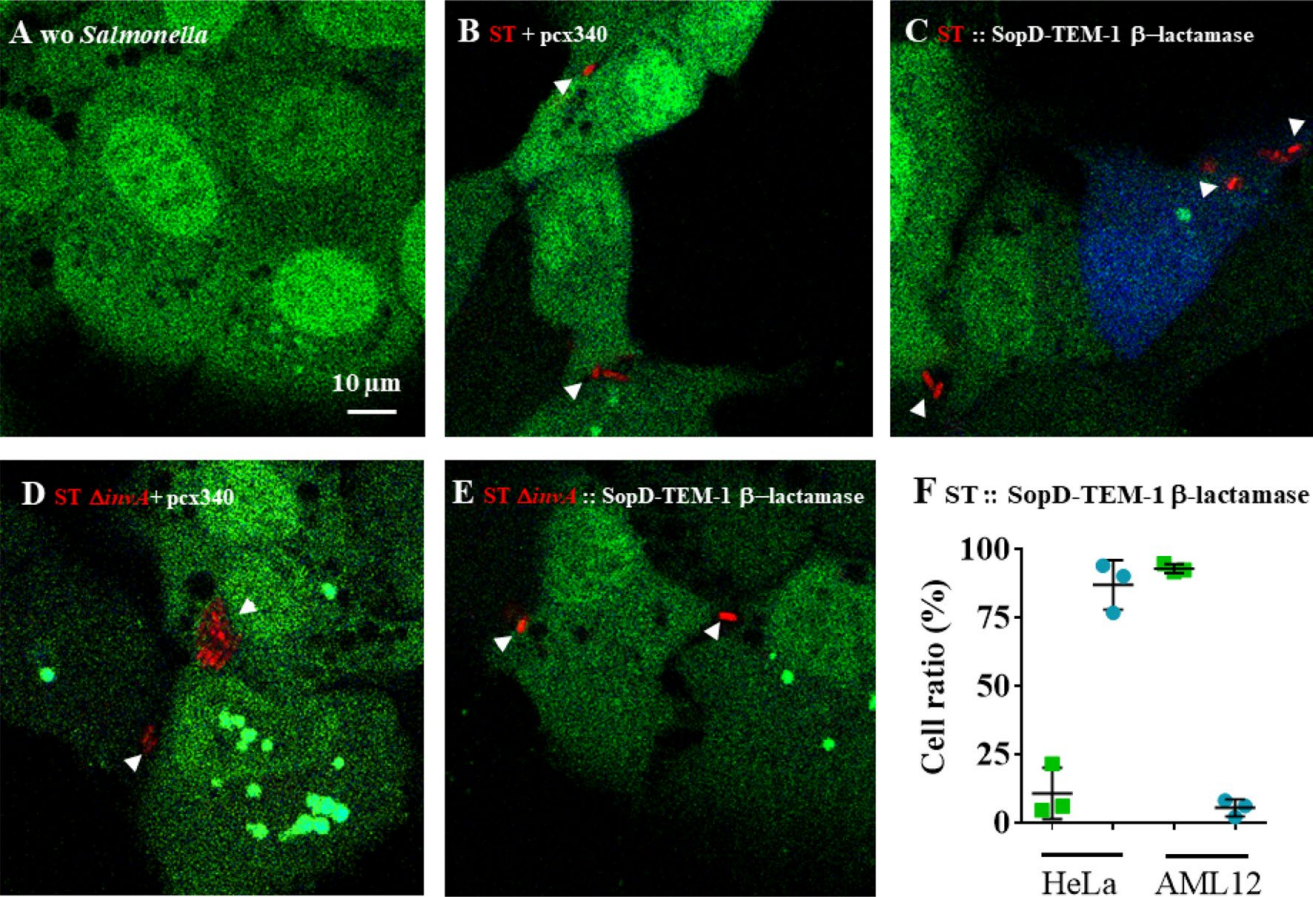
T3SS1 effector translocation occurred only in very few infected AML12 cells. AML12 cells were exposed for 30 min to ST or ST Δ*invA* strains expressing SopD effector-TEM-1 β-lactamase in pCX340 and DsRed in pGG2, then washed and infection was continued for 60 min. Subsequently, cells were loaded with CCF4-AM for 20 min and visually inspected (**A**–**E**) using an inverted confocal microscope equipped with a 100 × oil immersion objective (LEICA TCS SP8, Germany). Images of 1024 × 1024 pixels were acquired using LaserX software (LEICA). Green fluorescence indicates that CCF4-AM was loaded and the presence of blue cells due to CCF4-AM cleavage reveals SopD translocation. White arrowheads indicate *Salmonella*. (**A**) non-infected AML12 cells, (**B**) cells infected with the ST DsRed strain carrying the empty vector pCX340, (**C**) cells infected with the ST DsRed strain expressing SopD effector-TEM-1 β-lactamase in pCX340, (**D**) cells infected with the ST Δ*invA* DsRed strain carrying the empty vector pCX340 and (**E**) cells infected with the ST Δ*invA* DsRed strain expressing SopD effector-TEM-1 β-lactamase. The cells emitting blue fluorescence were considered as positive for effector translocation. (**F**) HeLa and AML12 cells were infected as described above with the ST strain expressing SopD effector-TEM-1 β-lactamase and DsRed, CCF4-AM loaded and finally trypsinized. Cell resuspensions were acquired using cytometry (BD LSRFortessa X-20 cell analyzer) and analyzed by BD FACSDiva Software. Green squares represent cells infected without SopD translocation; Blue dots represent infected cells with SopD translocation. Scale bars 10 µm.

The fact that a Δ*invA* mutant strain was as invasive as the wild-type strain in AML12 cells raised the question of the putative role of T3SS1 effectors in the invasion and in the early maturation of the SCV, considering these cells were infected with the wild-type strain. Was the T3SS1 apparatus used or not? The T3SS1 apparatus is a needle complex that allows for the injection of bacterial T3SS1 effector proteins into host cells. Direct interaction of these effectors with host proteins leads to massive actin cytoskeleton recruitment resulting in large membrane rearrangements and engulfment of the pathogen. While invasion-receptor interactions induce weak membrane rearrangement. In order to determine how the wild-type and Δ*invA* strains of *S.* Typhimurium entered AML12 cells, we compared membrane rearrangements induced by ST and ST Δ*invA* in AML12 cells using scanning electron microscopy (SEM) (Fig. 2C,D) and transmission electron microscopy (TEM) (Fig. 2E,F). *S.* Typhimurium SL1344 was chosen as a positive control for the visualization of large membrane rearrangements in HeLa cells (Fig. 2A), because the absence of SopE and a slightly different regulation of SPI1 in *S.* Typhimurium 14028 does not allow for the visualization of the ruffles even if this strain enters using its T3SS1 in this cell line^26^. Filopodia were distributed homogeneously at the cell surface of non-infected AML12 cells. (Fig. 2B). Despite numerous acquisitions of AML12 cells infected from 10 to 120 min with ST or ST Δ*invA*, we never observed large membranous ruffles on the surface of AML12 cells regardless of which strain was used to infect cells. All captured images showed only weak membrane rearrangements (Fig. 2C,D). One inhibitor of each entry mechanism: zipper or trigger, was then used to confirm our electronic microscopy results. Zipper mediated internalization is known to depend on the PI3kinase pathway^6^. The PI3 kinase inhibitor wortmannin was used on HeLa and AML12 cells. Significant reduction in invasion for both the ST strain (*p* = 0.028) and the ST Δ*invA* strain (*p* = 0.083) were observed only in AML12 cells (Fig. 3A). Cytochalasin D is an inhibitor of actin filament function that impacts severely large membranous ruffles. The working solution of Cytochalasin D 1 µg/mL, significantly reduced the entry of the *Salmonella* wild-type strain into HeLa cells with a ratio of 21.42 (*p* < 0.0001), but had a weak or insignificant impact on AML12 cell invasion by ST or ST Δ*invA* strains; there was a decrease of invasion of 1.93 (*p* < 0.0311) and of 0.8 (*p* < 0.8819) respectively (Fig. 3B). These results, in addition to the electron microscopy results favor a zipper-like entry into AML12 cell line. The absence of a trigger mechanism induced by the wild-type strain could have been due to a non-functional T3SS1 after interaction with AML12 cells. To explore this hypothesis, we imaged T3SS1 effector translocation in AML12 cells using a fluorescence-based translocation assay (Fig. 4A–E). Using cytometry, we then quantified the percentage of infected cells in which effector translocation occurred (Fig. 4F). To this end, SopD, a T3SS1 effector, fused with TEM-1 β-lactamase was cloned into a pCX340 vector and transformed in ST or in ST Δ*invA* strains expressing DsRed. SopD translocation in AML12 cells was monitored using a CCF4-AM assay. AML12 cells infected with ST or its Δ*invA* derivative strain and harboring the empty vector pCX340, were used as negative controls. Using Fluorescence Resonance Energy Transfer (FRET) quantification, it is described that in absence of β-lactamase activity the intact CCF4 molecule results in FRET, emitting a green fluorescent signal. While, in the presence of β-lactamase activity, consistent with SopD translocation, cleavage of CCF4 produces a blue fluorescence signal. All the strains were able to invade this cell line. As predicted, only green fluorescence signals were obtained when AML12 cells were infected with the control strains (Fig. 4A,B,D). Moreover, no SopD translocation was observed when cells were infected with ST Δ*invA* (Fig. 4E). By contrast, we observed that ST was able to translocate SopD in some cells (Fig. 4C), but for most cells, ST was able to invade with no apparent SopD translocation. This result was confirmed using flow cytometry analysis. We found that SopD translocation did not occur in 93.1% of the AML12 cells invaded by the ST strain. However, 87.1% of the cells were positive for both intracellular *Salmonella* and SopD translocation when HeLa cells were used as a control (Fig. 4F).

In conclusion, the *S.* Typhimurium 14028 strain enters into AML12 cells independently of T3SS1. Nevertheless, a very low number of bacteria (< 7%) remain able to translocate T3SS1 effectors in this cell line. The T3SS1 effector translocation observed in AML12 cells demonstrated that the T3SS1 apparatus is functional and can inject effectors within the host cells.

### In AML12 cells, *Salmonella* Typhimurium does not induce passive bacterial entry in a macropinocytotic way

**Figure 5:**
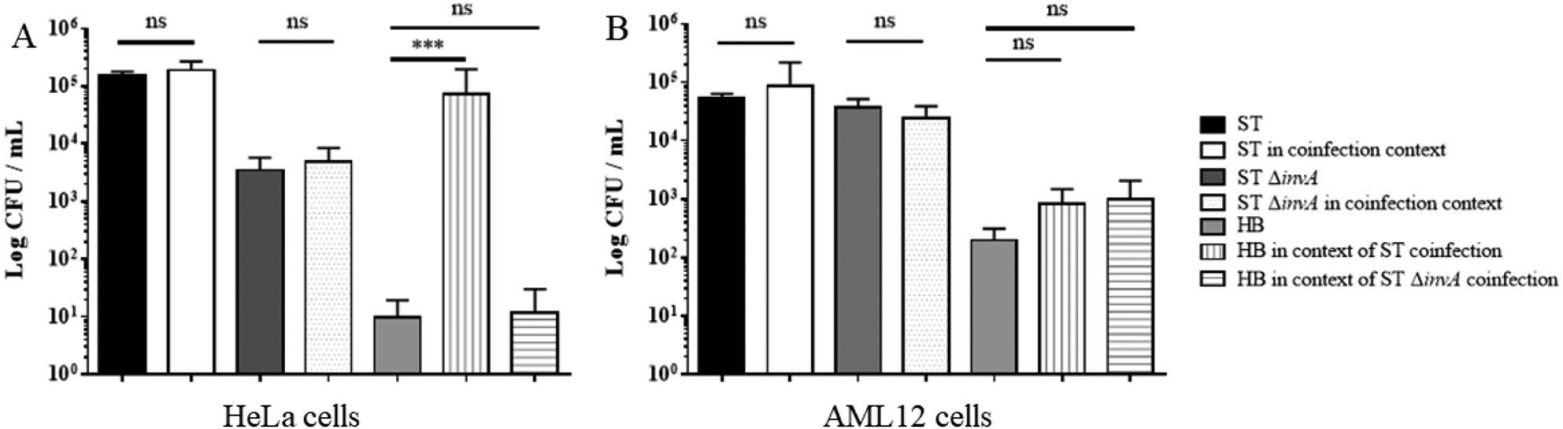
*Salmonella* Typhimurium vacuole maturation was mostly macropinosome independent in murine AML12 cells. HeLa cells (**A**) and AML12 cells (**B**) were grown in 24-well plates. Infection of cells was carried out for 60 min in 300 µL medium without serum at MOI 50 with ST or ST Δ*invA* alone or coinfected with the *E. coli* HB101 strain transformed with pSUP202, allowing for chloramphenicol selection. To quantify invasion, after 60 min of infection, cells were washed, and gentamicin protection assays were performed consisting of three washes with medium followed by 60 min in medium with 100 µg/mL gentamicin. After this, cells were extensively washed and lysed, and serial dilutions were plated on TSA and TSA chloramphenicol. Co-invasion was estimated by subtracting growth observed on TSA chloramphenicol to growth on TSA. The black bar corresponds to ST invasion without coinfection, the white bar ST invasion in coinfection context, the dark grey bar ST Δ*invA* invasion without coinfection, the dot bar ST Δ*invA* invasion in coinfection context, the light grey bar *E. coli* HB101 invasion without coinfection, the vertical line bar *E. coli* HB101 invasion in ST coinfection context, the horizontal line bar *E. coli* HB101 invasion in ST Δ*invA* coinfection context. Data are the means and standard deviations from three independent experiments. Statistical analyses using a Mann Whitney test were performed. Significance was (***) *p* = 0.0002.

Among endocytic mechanisms, macropinocytosis has been described as a mean of extracellular nutrient uptake. Several pathogens are known to induce this process during their entry into host cells allowing the passive entry of bacteria^27^. To further investigate the impact of the macropinocytosis in HeLa and AML12 cells, a coinfection experiment was performed. HeLa and AML12 cells were infected independently with ST or ST Δ*invA* or coinfected with ST plus *E coli* HB101 or ST Δ*invA* plus *E coli* HB101; *E coli* HB101 is non-invasive in HeLa cells and is a weakly invasive strain in AML12 cells (Supplementary Fig. 2). Our coinfection experiments had no impact on *Salmonella* invasion on either cell model. However, in HeLa cells, *E coli* HB101 invasion was significantly (*p* = 0.0002) increased when cells were coinfected with ST but not with ST Δ*invA* (Fig. 5A). This result can be explained by the fact that membranous perturbations induced during a trigger mechanism are associated with macropinocytosis inducing entry of a non-invasive strain in cooperation with an invasive strain^27^. On the other hand, in AML12 cells, *E coli* invasion was not increased in coinfection assays with ST or ST Δ*invA*, (Fig. 5B). Therefore, unlike in HeLa cells, this demonstrates that *Salmonella* invasion in AML12 cells is not associated with macropinocytosis.

### In AML12 cells, *Salmonella* Typhimurium 14028 resides intracellularly, mainly in a vacuole

**Figure 6:**
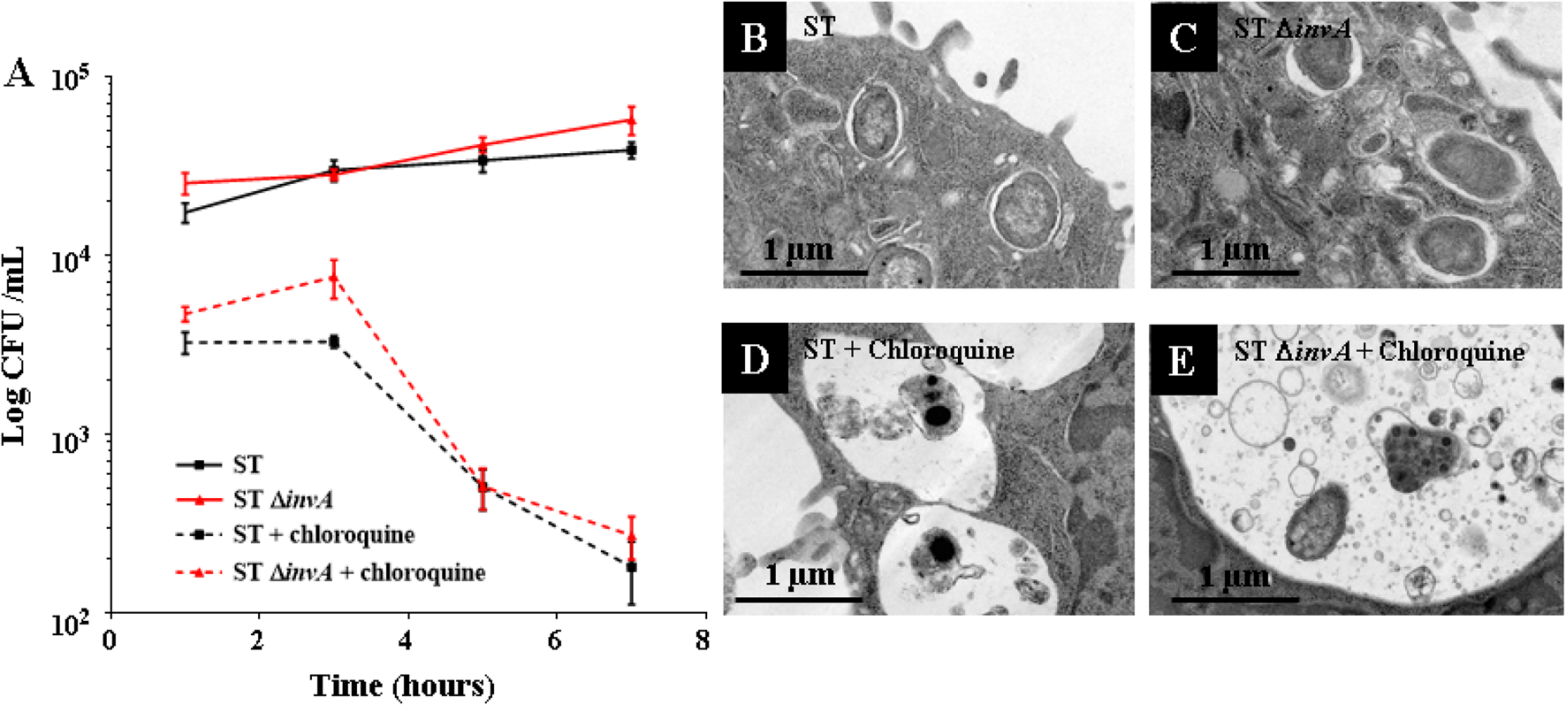
*Salmonella* Typhimurium resided in vacuoles after AML12 cell invasion. After 60 min exposure to ST (black squares) or ST Δ*invA* (red triangles) strains, AML12 cells were incubated with gentamicin at 100 µg/mL (solid lines) or gentamicin at 100 µg/mL plus 1.2 mM chloroquine (dashed lines) for 60 min and for 3, 5 and 7 h pi with gentamicin at 10 µg/mL (solid lines) or gentamicin at 10 µg/mL plus 1.2 mM chloroquine 60 min before the end point (**A**). At the 7 h timepoint, ST and ST Δ*invA* infected cells without (respectively **B**,**C**) or with chloroquine (respectively **D**,**E**) treatment were imaged using transmission electron microscopy (JEOL 1011, Tokyo, Japan). Scale bars 1 µm.

**Figure 7:**
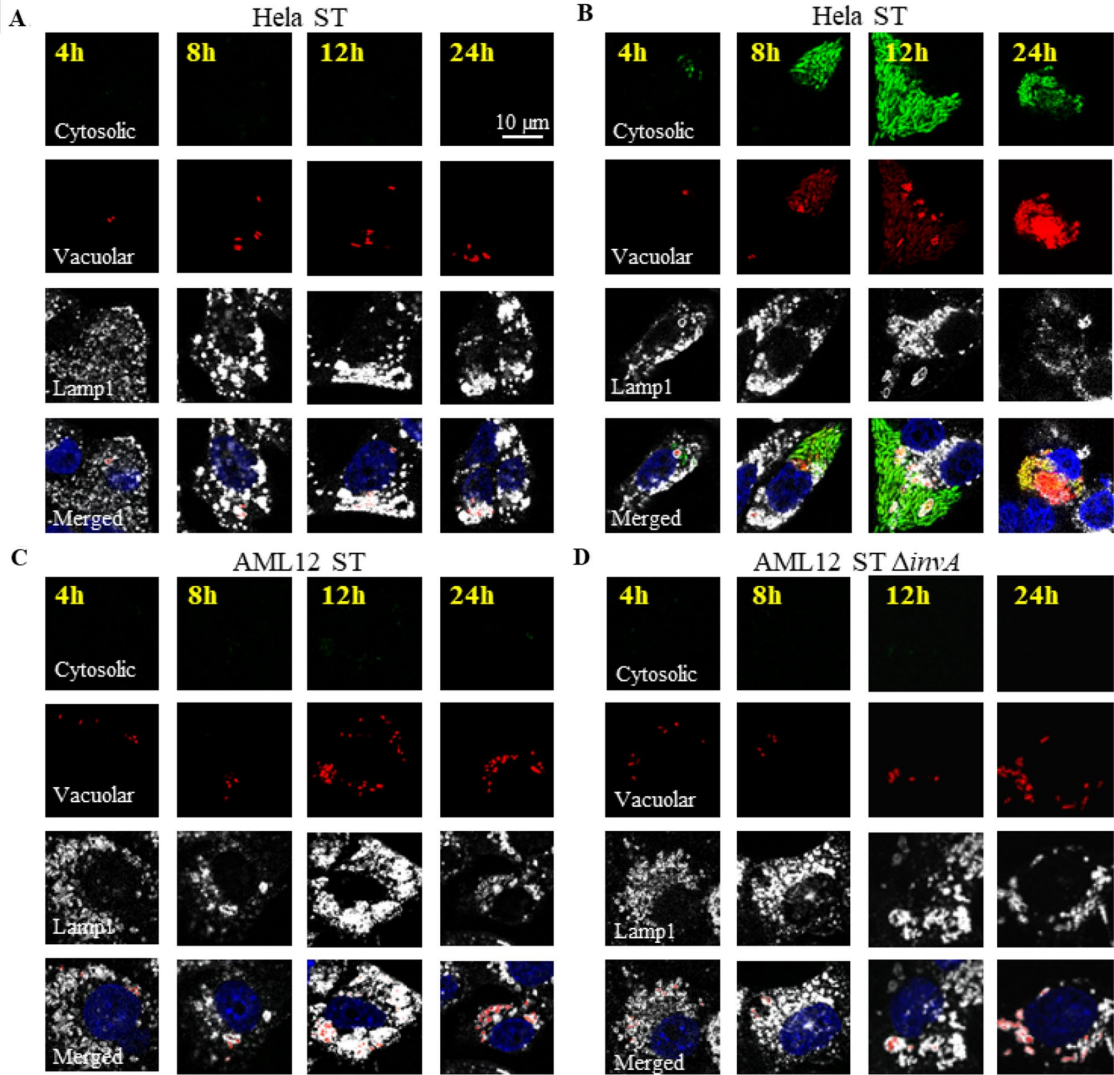
Cytosolic escape did not occur in AML12 cells contrary to Hela cells. HeLa cells (**A**,**B**) and AML12 cells (**C**,**D**) were exposed to ST p4889 (**A**–**C**) and ST Δ*invA* p4889 (**D**) 1 h followed by a gentamicin protection assay, then fixed and stained with mouse Lamp1 or rat Lamp1 antibody, respectively, then revealed with anti-mouse or anti-rat antibody Alexa Fluor 647 (white) imaged at 4, 8, 12, and 24 h pi. (**A**–**D**) are organized as a first line of images, allowing to potentially observe cytosolic bacteria (green) second line vacuolar and cytosolic bacteria (red), third line Lamp1 staining (white) and fourth line merged image of the first three lines (cytosolic bacteria appeared yellow to green). Imaging was performed using confocal microscopy with a 100 × oil immersion objective (Leica TCS SP8, Germany). Nuclei were counterstained with DAPI (blue) vacuolar bacteria (red) and cytosolic bacteria (green and weakly red = yellow to orange, mainly green due to hyper-replication). Scale bars 10 µm.

We then wished to determine where *Salmonella* is capable of replicating consecutively in a T3SS1-independent invasion. Indeed, cytosolic *Salmonella* constitutes a significant proportion of the total bacterial population in several epithelial cell models^20^. Over time, we followed the replication of *S.* Typhimurium in AML12 cells with or without chloroquine, as this molecule has been shown to kill intravacuolar *Salmonella*^20^. Compared to 1h post-infection (pi), we found that there were 2.76 and 2.62 times more bacteria at 7 h pi for the ST and ST Δ*invA* strains, respectively. Our results show that without chloroquine, the ST and ST Δ*invA* strains were able to survive and slightly multiply inside AML12 cells during the duration of the experiment. In contrast, a 95% reduction in the number of these two strains was observed when chloroquine was added (Fig. 6A). These results support the idea of an intravacuolar localization of *S.* Typhimurium in AML12 cells at 7 h pi, leading to a multiplication rate about 10 times for ST and ST Δ*invA* at 16 h pi (Supplementary Fig. 2 and Fig. 1). To confirm bacterial localization, we performed a TEM analysis at 7 h pi on AML12 cells infected with ST or ST Δ*invA* strains, treated or not with chloroquine. In the absence of chloroquine, we observed the presence of intact bacteria only in vacuolar space (Fig. 6B,C); bacteria appeared as expected damaged or degraded in vacuoles when cells were exposed to chloroquine (Fig. 6D,E). This confirmed the intravacuolar localization of *Salmonella* in AML12 cells. To further investigate this observation of vacuolar residence of *Salmonella* in AML12 cells, we compared the localization of *Salmonella* in HeLa and AML12 cells using a dual color reporter plasmid p4889 (P_EM7_::DsRed P_*uhp*T_::sfGFP) with combined expression of constitutive DsRed and cytosolic GFP^28^. At each time point imaged, we easily identified *Salmonella* that was found only in its SCV (red) for AML12 (Fig. C,D) and both cytosolic (red and green) (Fig. 7B) and vacuolar (red) for HeLa cells (Fig. 7A,B). We never observed cytosolic ST (Fig. 7C) or ST Δ*invA* (Fig. 7D) in AML12 cells.

Altogether, these results demonstrate a vacuolar localization of *S.* Typhimurium in AML12 cells.

### Maturation of the *Salmonella* containing vacuole leads to *Salmonella* replication with *Salmonella*-induced filament formation

**Figure 8:**
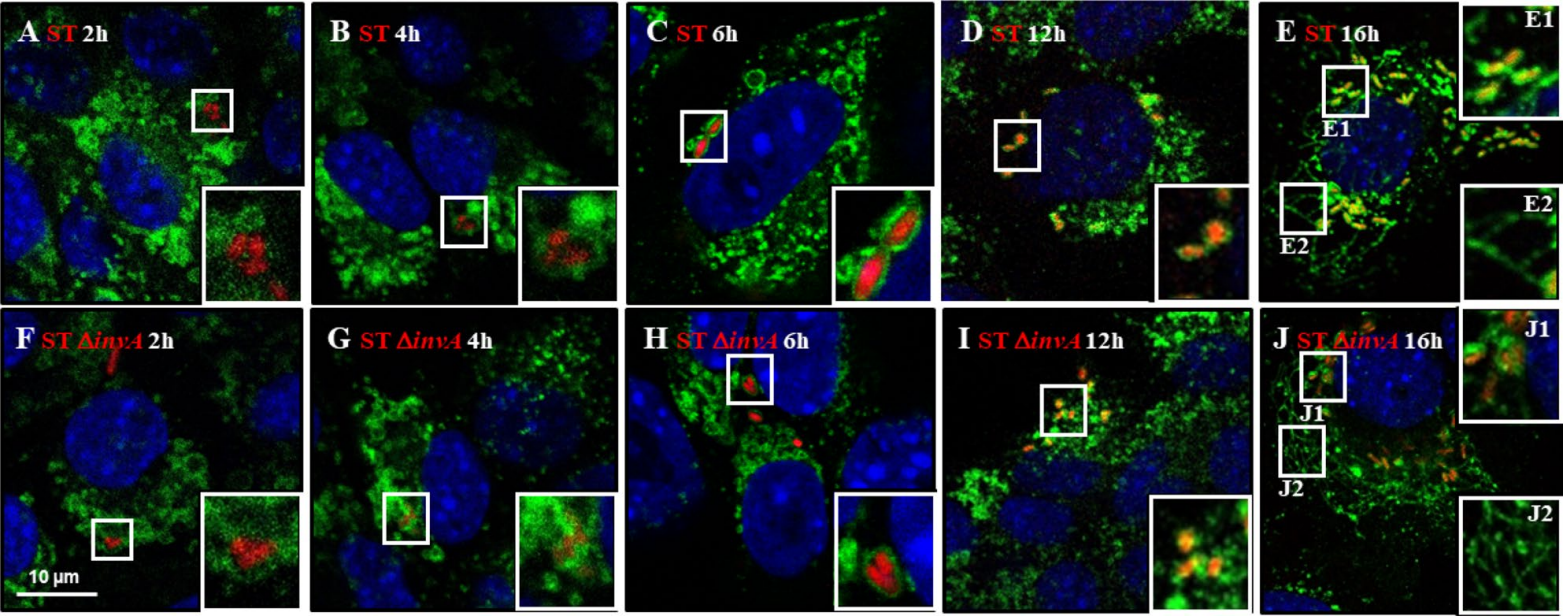
*Salmonella* containing vacuole maturation occurred in the presence of LAMP1 and led to SIF formation. The kinetics of LAMP1 recruitment was imaged for AML12 cells infected with ST DsRed and ST Δ*invA* DsRed at (**A**,**F**) 2 h, (**B**,**G**) 4 h, (**C**,**H**) 6 h, (**D**,**I**) 12 h and (**E**,**J**) 16 h pi. At each time point, cells were fixed and labelled with rat LAMP1 antibody, revealed with anti-rat Alexa Fluor 488 (green) and imaged using confocal microscopy with a 100 × oil immersion objective (Leica TCS SP8, Germany). Nuclei were counterstained with DAPI (blue). Squares outlined in white are a 2 × manual magnification of selected regions to visualize progressive recruitment of LAMP1. White outlined squares E1, J1 focus on *Salmonella* stuck in LAMP1 positive vacuoles and E2, J2 focus on SIF-like structures. Scale bars 10 µm.

**Figure 9:**
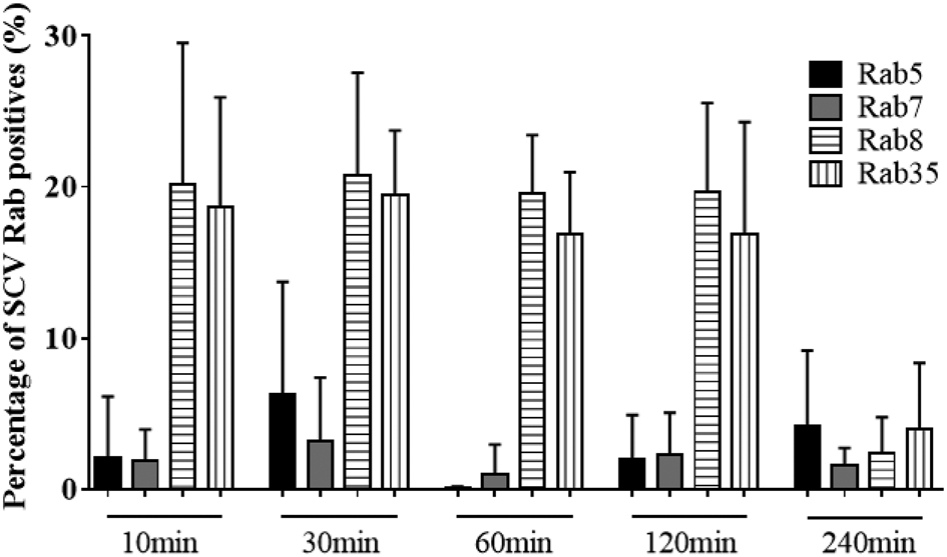
*Salmonella* containing vacuole associated with Rab GTPase markers. The localization of Rab GTPase to SCV from AML12 cells infected by ST DsRed at 10 min, 30 min, 60 min, 120 min and 240 min was estimated by labelling purified SCV with (black bar) mouse Rab5 antibody, (grey bar) mouse Rab7 antibody, (horizontal line bar) rabbit Rab8 antibody or (vertical line bar) rabbit Rab35 antibody revealed with anti-mouse Alexa Fluor 488 and anti-rabbit Alexa Fluor 488, respectively. Acquisition of SCV events were discriminated from PNS based on DsRed fluorescence using cytometry (BD LSRFortessa X-20 cell analyzer) and analyzed using FCS Express 4 Flow. Data are the means and standard deviations from three independent triplicates experiments.

SCV maturation has been deeply scrutinized in T3SS1-dependent invasion models. It is widely accepted that it consists of three stages: early, intermediate and late^29^. To interpret SCV maturation after T3SS1-dependent invasion, we exposed AML12 cells to ST or ST Δ*invA* strains for 1 h. In this way, we investigated the recruitment of several host proteins as markers of the different stages^14,17,30^, as well as the formation of SIF-like structures and the acidification of the vacuole^18,31^. Following immunocytochemistry using antibodies raised against Rab5, Rab7, Rab8 and Rab35 at 60 min (Supplementary Fig. 3) and raised against LAMP1 at 2, 4, 6, 12 and 16 h pi (Fig. 8), cells were imaged using confocal microscopy. Neither strain showed a high recruitment or a convincing colocalization of the Rab GTPases tested with intracellular *Salmonella.* Only a few dots near intracellular *Salmonella* were observed (Supplementary Fig. 3). However, as early as 4 h pi, we observed a slight recruitment of LAMP1 that became undeniable at 6, 12 and 16 h pi (Fig. 8). Moreover, SIF-like structures were clearly identified at 16 h pi (Fig. 8E2,J2) in 50.33% and 51.67% of ST and ST Δ*invA* infected cells, respectively (Supplementary Fig. 4). These results are in accordance with the proportion of SIF observed in HeLa cells for comparable multiplicity of infection (MOI) and cell density^32^. Having the goal to estimate Rab GTPase recruitment during SCV maturation, AML12 cells were infected with ST or ST Δ*invA*, and SCV were purified at 10, 30, 60, 120, 240 min and immunostained using antibodies raised against Rab5, Rab7, Rab8 or Rab35 and analyzed by flow cytometry. The example of Rab8 is demonstrated in Supplementary Fig. 5. At each time point, the percent positive SCV for Rab5 or Rab7 was less than 7% and the percent positive SCV for Rab8 or Rab35 was near 20% decreasing to less than 2% at 4 h pi (Fig. 9), thus confirming a low recruitment of these Rab GTPases to the SCV.

**Figure 10:**
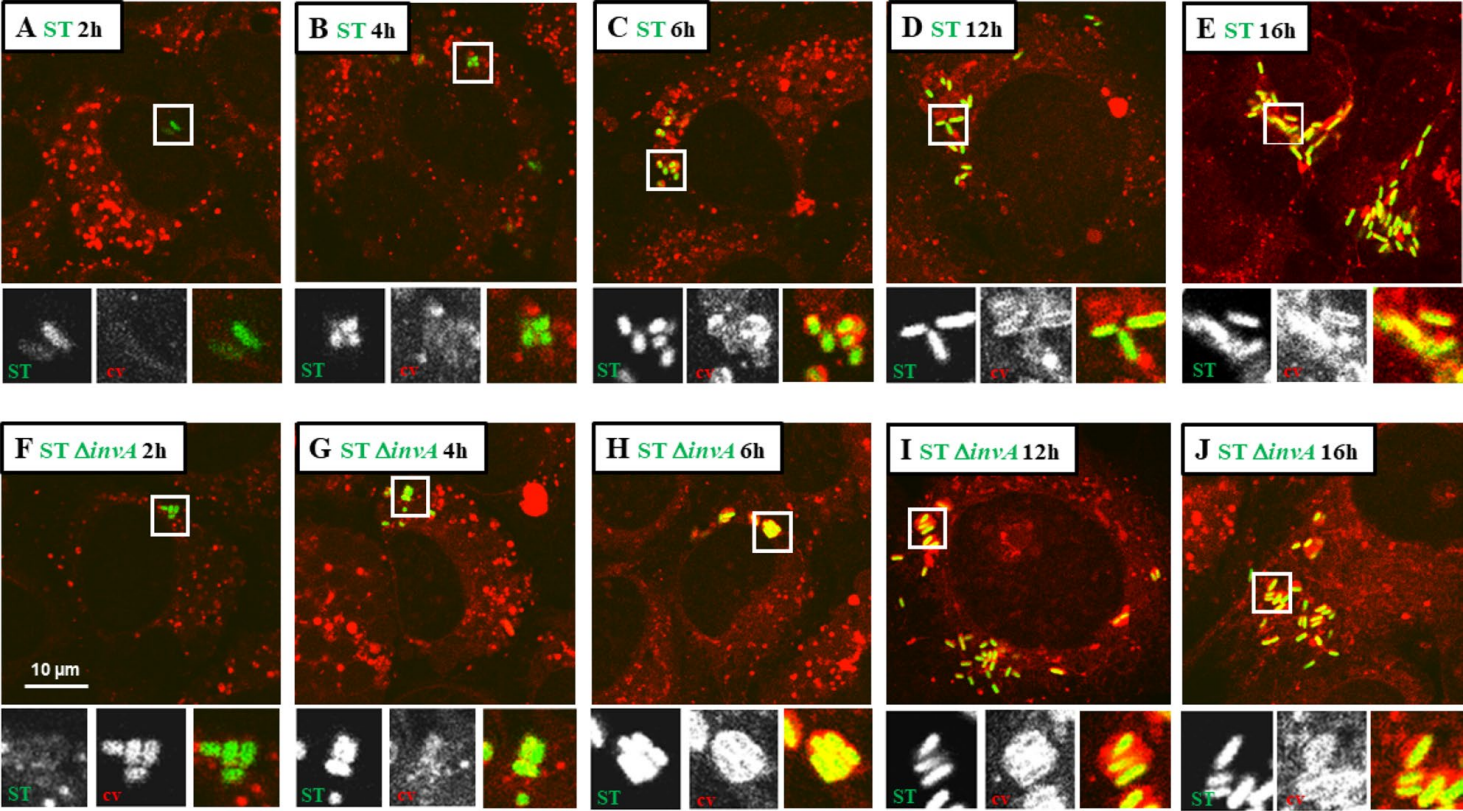
*Salmonella* Typhimurium progressed in acidified organelles in AML12 cells. The kinetics of vacuole acidification was processed for AML12 cells infected with ST GFP and ST Δ*invA* GFP at (**A**,**F**) 2 h, (**B**,**G**) 4 h, (**C**,**H**) 6 h, (**D**,**I**) 12 h and (**E**,**J**) 16 h pi. At each time point after 5 min exposure to 1 µM cresyl violet (cv) (red), cells were directly imaged using confocal microscopy with a 100 × oil immersion objective (Leica TCS SP8, Germany). Squares outlined in white are a 2 × manual magnification of select regions so as to visualize progressive organelle acidification. Scale bars 10 µm.

Previous studies have demonstrated that recruitment of LAMP1 and the presence of SIF-like structures depend on T3SS2 effectors when *Salmonella* enters host cells using its T3SS1^33^. Since T3SS2 is expressed in an environment with low pH as well as a low concentration of divalent cations, we tested whether or not the SCVs were acidified in the AML12 cells. Cresyl violet was used, as it is a sensitive and convenient acidotrophic fluorescent stain for acidified cell compartments^34^. To validate this tool for use in the study of SCV acidification, murine macrophages RAW 264.7 were infected with ST for 10, 30, 60, 120, 240 min and loaded with cresyl violet (Supplementary Fig. 6A to E, respectively). As expected, as early as 30 min pi, 35% of SCVs were acidified (Supplementary Fig. 6F) and this percentage continued to increase until reaching 89% 4 h pi, demonstrating that cresyl violet can be used to monitor SCV acidification. In parallel, AML12 cells were infected with ST and ST Δ*invA* for 2 h to 16 h pi and loaded with cresyl violet in order to determine if SCV acidified during AML12 infection (Fig. 10A–J and Supplementary Fig. 7A–C). At 4 h pi, 50% of the wild-type strains were embedded in an acidified vacuole. In contrast, at 16 h, there were over 90% (Supplementary Fig. 7A and C). Moreover, we observed that the vacuole acidification followed the same time-course as LAMP1 recruitment. Similar results were obtained when AML12 cells were infected with the *invA* mutant strain (Fig. 10F–J and Supplementary Fig. 7B and C).

**Figure 11:**
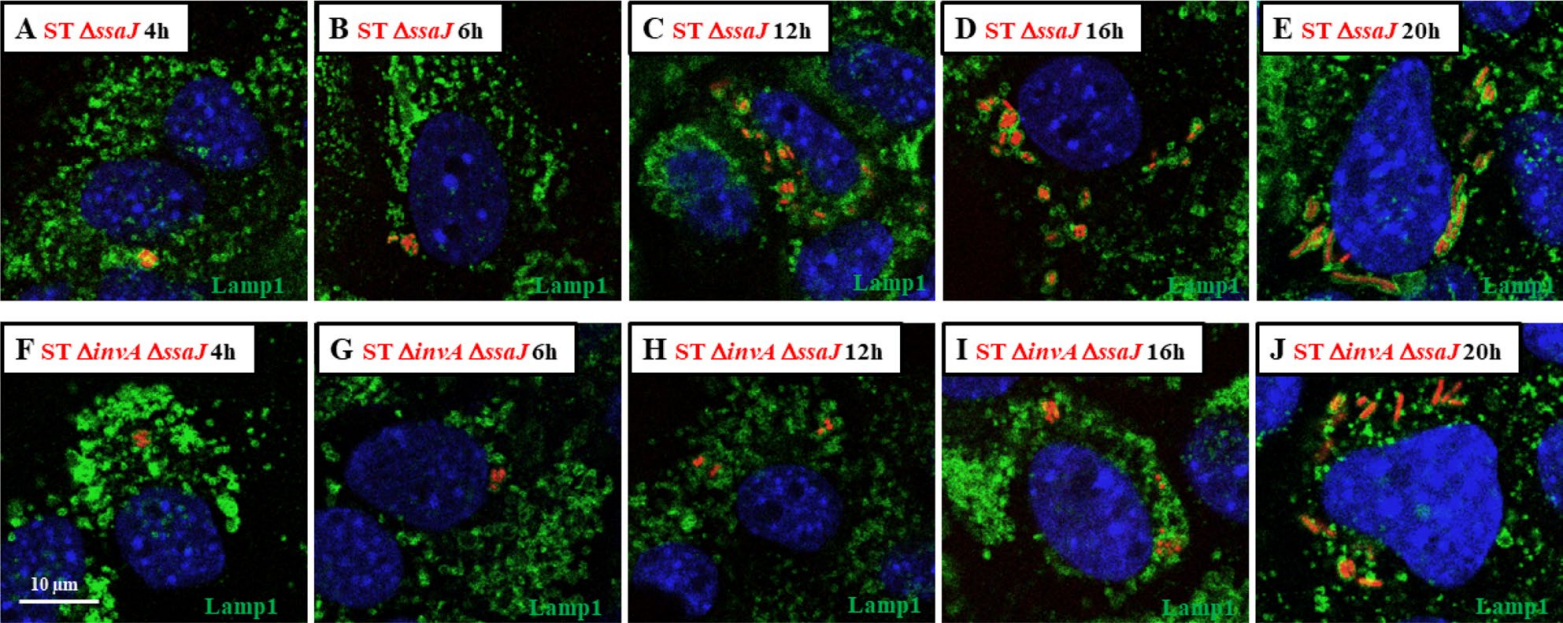
T3SS2-invalidated *Salmonella* Typhimurium resided in a vacuole in the presence of LAMP1 without SIF formation. The kinetics of LAMP1 recruitment was imaged for AML12 cells infected with ST Δ*ssaJ* DsRed and ST Δ*invA* Δ*ssaJ* DsRed at (**A**,**F**) 4 h, (**B**,**G**) 6 h, (**C**,**H**) 12 h, (**D**,**I**)16 h and (**E**,**J**) 20 h pi. At each time point, cells were fixed and labelled with rat LAMP1 antibody, revealed with anti-rat Alexa Fluor 488 (green) and imaged using confocal microscopy with a 100 × oil immersion objective (Leica TCS SP8, Germany). Nuclei were counterstained with DAPI (blue). Scale bars 10 µm.

Finally, we exposed AML12 cells to ST Δ*ssaJ* (lacking T3SS2) or to ST Δ*invA *Δ*ssaJ* (lacking T3SS1 and T3SS2) strains for 1 h and observed the recruitment of LAMP1 at 4, 6, 12, 16 and 20 h pi, as well as SIF formation. Unsurprisingly, for both strains, recruitment of LAMP1 in the SCV was detected between 4 and 12 h pi. However, at 16 h pi or longer, no SIF formation was seen (Fig. 11A–J). These results support the fact that there was no translocation of T3SS2 effectors due to *ssaJ* invalidation and also confirm that SIF formation observed with the wild-type strain was due to T3SS2 effectors.

Overall, these results demonstrate that after a T3SS1-independent entry in AML12 cells, *Salmonella* resides in a vacuole whose early maturation does not require the strong recruitment of Rab GTPase markers, as observed during a T3SS1-dependent entry^14,30^. Indeed, few SCVs were positive for Rab5 and Rab7 during the first 4 h of infection. After 2 h of infection, the percentage of SCV positive for Rab8 or Rab35 decreased to less than 5%. In contrast, the maturation of the vacuole appears later, similar to the two entry mechanisms with an acidification of the vacuole, LAMP1 recruitment and SIF-like structures formation. These observations were valid for both ST and ST Δ*invA* strains. In the absence of a T3SS1-mediated entry, *Salmonella* succeeded in retaining an SCV which favored its multiplication.

## Discussion

The interaction between *S.* Typhimurium and host cells has been explored in depth. Extensive knowledge exists about the crucial role of T3SS1 effector proteins in *Salmonella* invasion and intracellular behavior. These bacterial effectors interact with host cytoskeleton proteins allowing rearrangements in the host cell membrane. This leads to SCV formation, the maturation of which requires controlling of endocytic pathways using T3SS1 and T3SS2 effectors. This paradigm appears to be true for the majority of cell lines because among the non-phagocytic cell models that were previously tested, most were invaded by *Salmonella* via the T3SS1-dependent pathway^9,35,36^. This is the case for HeLa cells, which are often used to interpret *Salmonella* behavior^37^, but also for human colonic epithelial cell lines T84, and HT29^35^ as well as the alveolar murine epithelial cell MLE-12 line^38^. However, *Salmonella* can also enter non-phagocytic cell lines without its T3SS1. This is the case for fibroblastic cells^6^ or HT29 cells when these cells are cultured in 3D^39^. Similarly, Caco-2 cells, a partially T3SS1-independent cell model, become clearly T3SS1-independent in a co-culture model mimicking M cells^40^. In addition, the culture conditions of *Salmonella* also influence *Salmonella*-host cell interaction in vitro^41^. Consistent data illustrate the ability *of Salmonella* to use bacterial factors other than the T3SS1 to conserve their invasive power. Currently, Rck and PagN two membrane proteins of *Salmonella*, which are only slightly expressed in classic culture conditions, have been shown to be involved in the host cell invasion process^8,42^. Moreover, in a recently published study, we identified a panel of cells that could be invaded by a mutant devoid of all three known invasion factors at the same level as its wild-type counterpart^9^. This result clearly shows that further research is required in identifying new invasion factors.

In this context of multiple *Salmonella* invasion factors, we aimed to investigate whether the T3SS1 was required for intracellular *Salmonella* survival in the cell models described as T3SS1-independent. To determine this, we selected the AML12 murine hepatocyte cell line described as a T3SS1-independent cell model^9^. In all these experiments we decided to use ST and ST Δ*invA* grown overnight, a culture condition that weakly induces the expression of Rck and PagN (Supplementary Fig. 1). Firstly, we confirmed that ST and ST Δ*invA* were able to invade, survive and multiply at the same level in the AML12 cell line. Electronic imaging analysis revealed only weak membrane rearrangements consistent with the zipper mechanism (Fig. 2) and a low level of cells (5.5%) containing translocated T3SS1 effectors (Fig. 4). This supporting the fact that the T3SS1 apparatus was functional but not used in the invasion mechanisms of the murine AML12 hepatocytes in contrast to human HeLa adenocarcinoma epithelial cells. In addition to being involved in invasion process, T3SS1 is required for escape, survival and hyper-replication into the cytosol of cells where entry is T3SS1-dependent^43,44^. In the present study, using AML12 cells *Salmonella* resided in a SCV following invasion and, ST as ST Δ*invA* behaved in the same manner. Compared to results from the hyper-replicative model HeLa, C2BBe1, HuTu80 or HCT116 cells, no cytosolic replication occurred in AML12 cells^20^. The absence of *Salmonella* hyper-replication in the AML12 cytosol may explain the low rate of *Salmonella* at 7 h pi (Fig. 6). However, this low multiplication rate of 7 h pi is offset by the multiplication of *Salmonella* in the SCV observed at 16 h pi (Supplementary Fig. 2). Nevertheless, it would be interesting to explore whether or not the AML12 cellular context is in favor of non-growing intracellular bacteria, already described as persisters in macrophages^45,46^. This would be another way to explain the low rate of *Salmonella* multiplication observed at 7 h pi in AML12 cells. In fact, our work highlights an aspect of an epithelial cell type that promotes bacterial invasion and survival regardless of T3SS1 effectors. The rare translocation of T3SS1 effectors in host cells was not due to *Salmonella* culture conditions; the same inoculum had a marked opposite effect on HeLa and AML12 cells.

The steps of SCV maturation in T3SS1-dependent cell models have been well defined and can be detailed in early, intermediary and late vacuoles. T3SS1 effectors contribute with T3SS2 effectors in recruiting molecular cell partners, including Rab GTPases, that enrich the vacuole and allow for its maturation. In contrast, after a T3SS1-independent entry process the intracellular behavior of *Salmonella,* the nature of the vacuole and the requirements for vacuole maturation needed to be explored. Our chloroquine assays showed that ST resided only in the vacuoles of AML12 cells. Survival in this vacuole is T3SS1-independent. Indeed, we observed the same intravacuolar localization (Fig. 8) and the same multiplication rate for ST and ST Δ*invA* in AML12 cells, which is about 10 times. Moreover, we demonstrated the absence of recruitment of endocytic markers such as Rab5 and Rab7 in AML12 cells, which are used to characterize the SCV in HeLa cells^14,47^. This result was in accordance with the results obtained using the *invA* mutant as the recruitment of endosomal markers depends on their direct or indirect interaction with T3SS1 effectors^14,48^. This is not the first time that a SCV without Rab5 or Rab7 recruitment has been observed. Indeed, in HeLa cells infected with ST Δ*invA* strain expressing the invasin Inv of *Yersinia* allowing *Salmonella* invasion, it has been shown that Rab GTPase recruitment on the SCV is drastically different from that observed for the ST strain. For example, these vacuoles are not enriched with Rab5 and are poorly enriched with Rab7, but seem more enriched with Rab8 and Rab35^30^. In contrast, we could clearly observe vacuole recruitment of the late endosomal marker LAMP1 with both ST and ST Δ*invA* strains. Similarly, whichever the strain considered, we observed the formation of SIF-like structures, but later (16 h) than for T3SS1-dependent models (8–12 h). These results confirm the maturation of the SCV even though early endosomal markers were not highly recruited. One can either hypothesize that *Salmonella* in the vacuole waits for the natural acidification of the phagosome to initiate the T3SS2 dialogue, or that a signaling cascade requiring other bacterial and cellular actors, allowing *Salmonella* to survive in a mature vacuole, is involved and remains to be identified. In actual fact, it was reported that vacuolar constraints stimulate the PhoP-PhoQ system and influence gene transcription like *pagN*, potentially implicated in *Salmonella* intracellular fate^49^.

In conclusion, *Salmonella* can enter, survive and multiply in a SCV without using its T3SS1, as demonstrated in this paper using the AML12 cell line. This interaction with host cells does not lead to hyper-replication in the cytosol, and the maturation of the SCV follows a different pathway than that described to date. We are currently investigating whether or not this process is universal for other cell lines (*e.g.* for which a T3SS1-independent invasion has already been observed). Moreover, host cell proteins and *Salmonella* partners implicated in the SCV maturation in this context remain to be identified.

## Materials and methods

### Generation and maintenance of *Salmonella* strains

*Salmonella enterica* serovar Typhimurium ST 14028 (ST) for which we previously demonstrated the T3SS1 independent entry into AML-12 cells was the wild-type strain used in this study. The T3SS1 deficient strain *S.* Typhimurium Δ*invA* (ST Δ*invA*) and the T3SS2 deficient strain *S.* Typhimurium Δ*ssaJ* were obtained by deleting the *invA* or the *ssaJ* open reading frame respectively, using the λ Red recombinase method^50^ previously described^10^. Briefly, the open reading frame was stably replaced by a kanamycin marker, and successful deletion/insertion of the target gene was confirmed using PCR analyses, and the kanamycin cassette was removed. DsRed and GFP expressing strains used in this study were obtained by bacterial electroporation of plasmid pGG2-DsRed^51^ and pFPV25.1 GFP^52^. For infection, bacteria expressing pGG2-DsRed or pFVP25.1 GFP were grown in TSB with 10 µg/mL carbenicillin 8 h at 37 °C with shaking, diluted 1:100 in fresh TSB, and incubated overnight at 37 °C without shaking. Bacteria were diluted 1:5 and absorbance was measured. Subsequently, bacteria were added to the cells at the required multiplicity of infection (MOI) in medium without fetal calf serum. *Salmonella enterica* serovar Typhimurium SL1344 grown using the same conditions as for ST, was used as control known to induce a trigger mechanism imaged by MEB.

### Invasins expression profiles

For the Rck and PagN invasins expression study, *Salmonella* Typhimurium 14028 BamB::3xFLAG or PagN::3xFLAG were obtained (as already described for *Salmonella* Typhimurium 14028 Rck::3xFLAG^24^) and were grown overnight in TSB. Then, 10 µl of a 10^10^ bacteria/mL suspension in Laemmli buffer were loaded after heat denaturation at 100 °C on 4–15% Mini-PROTEAN® TGX™ Precast Protein Gels. Transfer was performed using a nitrocellulose membrane, and blots were stained with rabbit anti-Flag M2 or homemade rabbit anti-BamB^53^. This was followed by incubation with anti-rabbit HRP revealed with SuperSignal West Dura from Thermofisher and acquired using FX Fusion (Vilber Lourmat).

### Cell culture

The murine AML12 hepatocytes (ATCC CRL-2254) are a spontaneously immortalized cell line from mice transgenic for transforming growth factor alpha. AML12 was identified as a cell model of T3SS1-independent invasion. Cells were routinely grown in 75 cm^2^ plastic tissue culture flasks at 37 °C under 5% CO_2_ in DMEM-F12 cell culture media with 0.005 mg/mL insulin, 0.005 mg/mL transferrin, 5 ng/mL selenium, 40 ng/mL dexamethasone and 10% fetal bovine serum, without antimicrobial compounds. HeLa cells (ATCC CRM-CCL-2), used as a reference for a T3SS1-dependent cell model, were grown in DMEM with 2 mM L-Glutamine and 5% fetal bovine serum^9^.

### Adhesion, invasion, multiplication assays

*Salmonella* adhesion, invasion and multiplication assays were performed with sub-confluent cell monolayers seeded in 24-well plates. Infection of cells was carried out for 60 min in 300 µL of medium without serum at MOI 50. For the adhesion/invasion assay, five washes in buffered saline water were followed by cell lysis in 4 °C H_2_O using serial dilution plated on Tryptic Soy Agar (TSA-DIFCO). To quantify for invasion only, after 60 min of infection, cells were washed, and gentamicin protection assays were performed consisting of three washes with medium, followed by 60 min in medium with 100 µg/mL gentamicin. After this, as for the adhesion/invasion assay, cells were extensively washed and lysed, and serial dilutions were plated on TSA. Following the gentamicin protection assays, gentamicin multiplication assays were performed. Cells were washed three times with medium followed by a further 16 h in medium with 10 µg/mL. Subsequently, as for the adhesion/invasion assay, cells were extensively washed and lysed, and serial dilutions were plated on TSA.

### Scanning and transmission electron microscopy

HeLa and AML12 cells were cultured at 10^6^ cells per well 48 h before infection on glass coverslips (diameter 12 mm) in 24-well plates for scanning electron microscopy, and then directly in wells for transmission electron microscopy. Cells were infected with *Salmonella* strains at MOI 50 for 30 min in HeLa cells, trigger mechanism control and for 15, 30, 45 or 60 min in AML12 cells.

Scanning electron microscopy samples were carried out using infected cells fixed after incubation in 4% paraformaldehyde, 1% glutaraldehyde (SIGMA, St-Louis, MO) in 0.1 M phosphate buffer (pH 7.2) for 24 h. After washing in PBS, samples were fixed with 2% osmium tetroxide for 1 h. A graded series of ethanol solutions allowed for full dehydratation of cells, then samples were dried in hexamethyldisilazane (HMDS, SIGMA). Samples were coated with 40 Å platinum, using a GATAN PECS 682 apparatus (Pleasanton, CA). Observations were performed using a Zeiss Ultra plus FEG-SEM scanning-electron microscope (Oberkochen, Germany).

Transmission electron microscopy were performed using infected cells resuspended after trypsinization, washed in phosphate-buffered saline (PBS) and fixed in 4% paraformaldehyde, 1% glutaraldehyde (SIGMA, St-Louis, MO) in 0.1 M phosphate buffer (pH 7.2) for 24 h. Samples were washed in phosphate-buffered saline (PBS) and post-fixed with 2% osmium tetroxide (AGAR SCIENTIFIC, Stansted, UK) for 1 h. Then, complete dehydration of samples was carried out in a graded series of ethanol solutions followed by propylene oxide.This was followed by an impregnation step with a mixture of (1∶1) propylene oxide/Epon resin (SIGMA). Samples were kept overnight in pure resin. Ultra-thin sections (90 nm) of embedded cells in Epon resin (SIGMA) polymerized 48 h at 60 °C, were collected using a Leica EM UC7 ultramicrotome (Wetzlar, Germany). Sections were stained with 5% uranyl acetate (AGAR SCIENTIFIC), 5% lead citrate (SIGMA) and were observed using a transmission electron microscope (JEOL 1011, Tokyo, Japan).

### T3SS1 effector translocation assays

β-lactamase reporter assays were performed as previously described^9,54^. SopD fused with β-lactamase TEM-1 was cloned in pCX340 plasmid. Recombinant plasmid was introduced either in ST DsRed or ST Δ*invA* DsRed. The resulting strains were used to infect AML12 cells for 30 min as described above, and 60 min in the presence of 1 mM isopropyl-β-d-thiogalactopyranoside (IPTG) to induce SopD-β-lactamase expression and in the presence of 1 mM probenecid. Post-infection, a small molecule substrate of β-lactamase (LiveBLAzer FRET-B/G Loading Kit with CCF4-AM, THERMOFISHER SCIENTIFIC) was added into the cell culture medium and incubated for 20 min at room temperature in the presence of 1 mM probenecid. Translocation of effectors fused to β-lactamase TEM-1 would result in the cleavage of CCF4 and emission of blue instead of green fluorescence using 405 nm laser excitation. The infected cells were visually inspected using an inverted confocal microscope equipped with a 100 × oil immersion objective (LEICA TCS SP8, Germany). Images of 1024 × 1024 pixels were acquired using LaserX software (LEICA). The cells emitting blue fluorescence were considered as positive for effector translocation. Flow cytometry analysis was performed with trypsinized cells from β-lactamase reporter assays described above. Analysis was carried out using 405 nm violet and 488 nm blue lasers to estimate the cleavage of CCF4-AM and 561 nm yellow/green laser for *Salmonella* pGG2-DsRed infected cells with a BD LSRFortessa X-20 cell analyzer. Twenty thousand infected cells were gated based on green/red (cells infected without cleaved CCF4) or blue/red (cells infected with cleaved CCF4).

### Invasion inhibition by drug assay

HeLa and AML12 cells were grown in 24-well plates preincubated with medium containing DMSO, wortmannin (100 nM) (SIGMA, St-Louis, MO) for 30 min, or with cytochalasin D (1 µg/mL) (SIGMA, St-Louis, MO) for 30 min followed by an infection and gentamicin protection assay with ST and ST Δ*invA* contining drugs.

### Macropinocytosis analysis through non-invasive *Escherichia coli* strain uptake

HeLa cells and AML12 cells were grown in 24-well plates. Infection of cells was carried out for 60 min in medium without serum at a MOI of 50 with either a *Salmonella* strain alone or with a *Salmonella* strain and also with *E. coli* HB101 or MC1061 strain transformed with pSUP202 allowing for chloramphenicol selection. To quantify invasion, after 60 min of infection, cells were washed and gentamicin protection assays were performed as described above. Serial dilutions were plated on TSA and TSA chloramphenicol 30 µg/mL. The latter medium allowed counting of *E. coli* while the number of *Salmonella* was obtained by substracting the number of colonies on TSA with the number of colonies on TSA chloramphenicol.

### Assessment of bacterial survival: vacuolar versus cytosolic

To evaluate whether or not multiplication occurred in a vacuole or in the cytosol, chloroquine resistance assays were performed. First, a serial dilution of chloroquine was carried out. The concentration of 1.2 mM chloroquine was estimated to be the most suitable to obtain the best vacuolar bacterial mortality without affecting AML12 cell survival and this was confirmed through a cell survival assay (Tetrazolium reduction assay (MTT) (Data not shown). After 60 min of contact between bacteria and cells, cells were incubated in growth media with gentamicin alone at 100 µg/mL or with the addition of chloroquine at 1.2 mM for 60 min. This was followed by lysis and plating, corresponding to 1 h pi. Afterwards, the same procedure was repeated at 3 h, 5 h and 7 h pi with gentamicin alone at 10 µg/mL or with the addition of chloroquine at 1.2 mM 60 min before the end point. In addition, at the 7 h timepoint, ST and ST Δ*invA* infected cells, chloroquine treated or not, were imaged using transmission electron microscopy (JEOL 1011, Tokyo, Japan).

### *Salmonella* cytosolic escape

To visualize bacterial escape from vacuoles, a dual color reporter plasmid p4889 (P_EM7_::DsRed P_*uhp*T_::sfGFP) was used^28^. P_EM7_::DsRed, allows for the expression of constitutive DsRed corresponding to vacuolar and cytosolic bacteria (red), P_*uhp*T_::sfGFP allows for inducible GFP expression in cytosol corresponding to cytosolic localization of bacteria (green). In confocal imaging, green and weakly red bacteria appear yellow to orange, mainly green due to *Salmonella* hyper-replication in the cytosol. HeLa cells were exposed to ST p4889 for 1 h, followed by a gentamicin protection assay. They were then fixed and stained with mouse Lamp1 antibody (Clone H4A3 from DSHB) and revealed with anti-mouse Alexa Fluor 647 (Thermofisher). AML12 cells were exposed to ST p4889 and ST Δ*invA* p4889 for 1 h followed by a gentamicin protection assay, fixed and stained with rat Lamp1 antibody (Clone 1D4B from DSHB), and then revealed with anti-rat Alexa Fluor 647 (Thermofisher). Images were obtained at 4, 8, 12, and 24 h pi. Imaging was performed using confocal microscopy (LEICA TCS SP8, Germany).

### Flow cytometry analysis of SCV

AML12 cells grown in P6 plates were infected with ST DsRed (MOI 100) 10 min, 30 min, 60 min in DMEM without SVF, 120 min (corresponding to an additional 60 min in complete medium gentamicin 100 µg/mL) and 240 min (corresponding to an additional 120 min in complete medium gentamicin 10 µg/mL). After infection, cells were washed in PBS 0.2% BSA and scraped in 3 mL of homogenization buffer HB (Sucrose 250 mM (SIGMA), Imidazole 3 mM pH7.4 (SIGMA), 0.1% gelatin (SIGMA), 0.5 mM EGTA (SIGMA), and Protease inhibitor cocktail (ROCHE). After centrifugation 5 min at 1800 g, pellets were resuspended in 400 µL of HB and gently homogenized by six passages through 22G syringe. After three centrifugations for 5 min at 100 g, 100 µL of supernatant containing post-nuclear supernatant (PNS) were labelled with mouse Rab5 antibody (SANTACRUZBIOTECH), mouse Rab7 antibody (SANTACRUZBIOTECH), rabbit Rab8 antibody (PROTEIN TECH) or rabbit Rab35 antibody (INVITROGEN) and revealed with anti-mouse or anti-rabbit Alexa Fluor 488 (INVITROGEN). Isotypic controls were done.

### Vacuolar acidification

For identification of acidified vacuoles, 300 µL of 2.10^5^ cell/mL suspension of RAW 264.7 cells or AML12 cells were seeded per well using 8 well chamber slide (Ibidi) 24 h before infection in complete medium. RAW264.7 cells were infected at MOI10 in complete medium with ST GFP 10, 30, 60, 120 and 240 min and incubated for 5 min with 1 µM cresyl violet at 37 °C. AML12 cells were infected with ST GFP or ST Δ*invA* GFP at MOI 50 and incubated for 5 min with 1 µM cresyl violet at 37 °C before image capture at 2, 4, 6, 12 and 16 h pi. Acidified SCVs were imaged. Three independent experiments were performed, and 100 infected cells were visually counted at each time point. Images were acquired using an inverted confocal microscope equipped with a 100 × oil immersion objective (LEICA TCS SP8, Germany), 488 nm and 585 nm laser excitation. Image detectors were set to collect signals emitted between 525/575 nm and 600/650 nm for bacteria and for cresyl violet in acidified cell vacuoles, respectively.

### Immunofluorescence and confocal microscopy

HeLa and AML12 cells were seeded in 24-well plates with 12 mm diameter glass coverslips at a density of 10^5^ cells per well, 24 h before infection, as previously described^9^. Cell infection was carried out using ST DsRed or ST Δ*invA* DsRed for 1 h at 37 °C in 5% CO_2_ at MOI 50. Cells were washed five times and exposed to 100 µg/mL gentamicin for 60 min, and when needed, to 10 µg/mL gentamicin for 4, 6, 12 16 and 24 h. After infection, cells were washed in PBS and fixed for 10 min with 4% formaldehyde in PBS at room temperature until selected end points: 2, 4, 6, 12, 16 and 24 h. Fixed cells were permeabilized with 0.1% saponin in PBS. Cells were immunostained with primary antibody anti-LAMP1 diluted 1/20 in PBS plus goat serum with 0.1% saponin (Supernatant culture from Clone 1D4B rat origin for AML12 cells and Clone H4A3 mouse for HeLa cells obtained from the DSHB, created by the NICHD of the NIH and maintained at The University of Iowa, Department of Biology, Iowa City, IA 52,242), washed in PBS with 0.1% saponin and immunostained with secondary antibody goat anti-Rat or anti-Mouse Alexa Fluor 488 or Alexa Fluor 647 diluted 1/400 in PBS plus goat serum 0.1% with 0.1% saponin. Cell nuclei were counterstained with DAPI. Glass slides were then coverslipped using in Fluoromount mounting medium^9^. Cells were observed using a SP8 confocal laser-scanning microscope equipped with a 100 × oil immersion objective (LEICA). Images of 1024 × 1024 pixels were acquired using LaserX software (LEICA). Sections 0.28 µm thick (38 per image) were assembled into Z stacks using Las AF lite 2.6.3 build 8173 software (LEICA). Figure 2 shows one representative section of the Z stacks.

### Statistical analysis

Experiments were repeated two to four times in triplicates or quadruplicates. Statistical analyses using a Mann Whitney test were performed using GraphPad Prism version 6.07 for Windows, GraphPad Software, La Jolla California USA, http://www.graphpad.com.

## Supplementary Information

Supplementary Information.
