# Supplementary material for: Murine AML12 hepatocytes allow Salmonella Typhimurium T3SS1-independent invasion and intracellular fate
Source: Sci Rep. 2021 Nov 23;11:22803. doi: 10.1038/s41598-021-02054-z (PMC8611075; doi:10.1038/s41598-021-02054-z)
Supplement: Supplementary file 1 — Supplementary Information. [file 41598_2021_2054_MOESM1_ESM.docx]

**
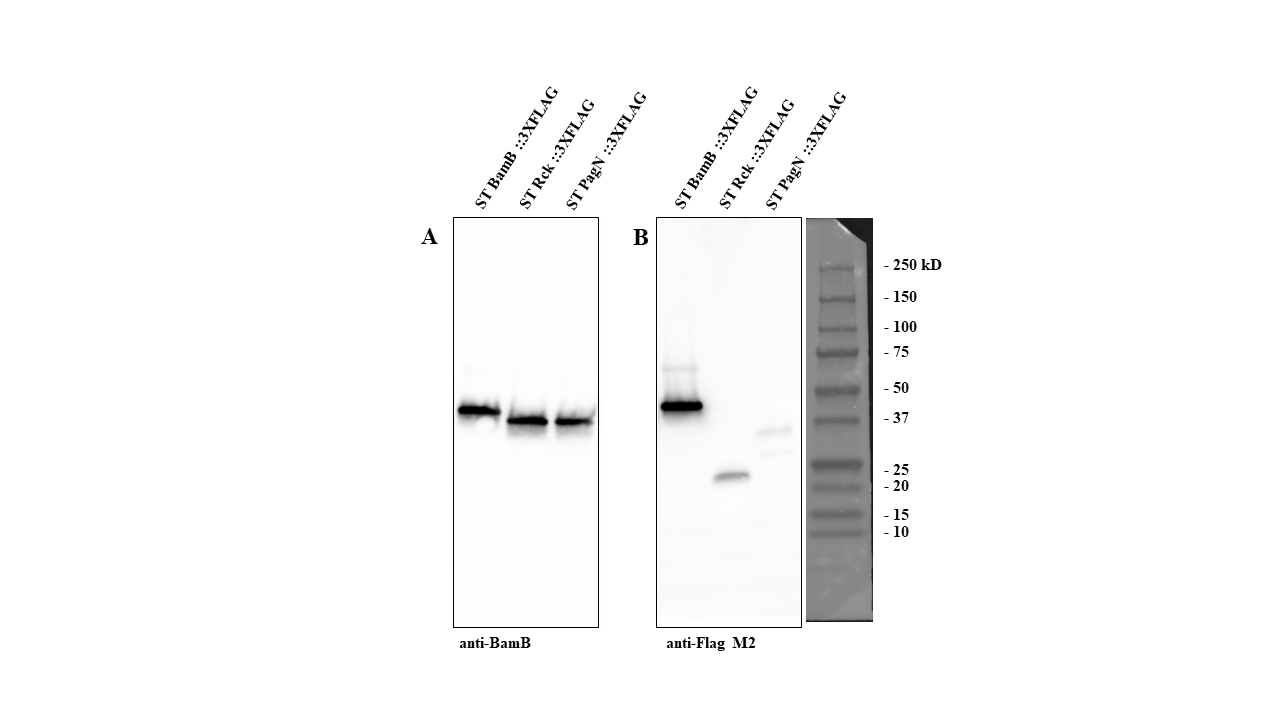
**

**Supplementary Figure 1:** **Low expression of Rck and PagN were observed after overnight culture in TSB medium.** *Salmonella* Typhimurium 14028 BamB::3xFLAG, Rck::3xFLAG or PagN::3xFLAG were grown overnight in TSB medium. Then 10^8^ bacteria suspended in Laemmli buffer were loaded in each well after heat denaturation at 100°C on 4–15% Mini-PROTEAN® TGX™ Precast Protein Gels. Transfer was carried out using nitrocellulose membranes stained with homemade rabbit anti-BamB (A) or rabbit anti-Flag M2 (B) followed by incubation with anti-rabbit HRP revealed with SuperSignal West Dura from Thermofisher and acquired using FX Fusion (Vilber Lourmat).


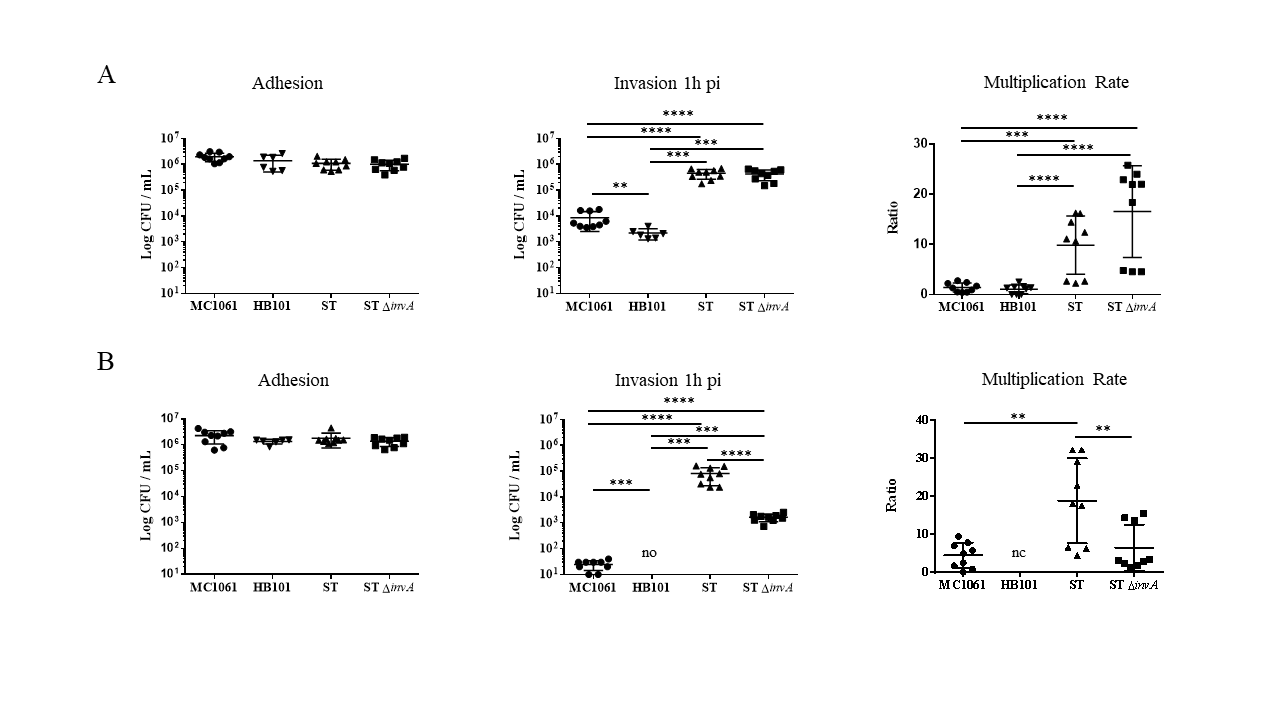


**Supplementary Figure 2: Invasiveness of *S.* Typhimurium 14028 in AML12 was not dependent on the permissiveness of this cell model.** In repeated gentamicin protection assays, bacterial adhesion, internalization 1 h pi and ratio 16 h pi over 1h pi in (A) the T3SS1-independent cell model AML12 and in (B) the T3SS1-dependent cell model HeLa were quantified for non-invasive *Escherichia coli* MC1061 (black dots) and *Escherichia coli* HB101 (black inverted triangles), and ST (black triangles) and ST Δ*invA* (black squares) strains. Cells were exposed to each strain (MOI = 50) for 60 min and washed three times (Adhesion) followed by the addition of gentamicin (100μg/mL) for 60 min and washed three times (Invasion), followed by the addition of gentamicin (10μg/mL) for 16 h (Multiplication). The results correspond to the mean ± standard deviation of at least two independent experiments performed in triplicate and expressed in log CFU/well. *E. coli* HB101 multiplication rate in HeLa cells is not calculable (nc) due to no-observed (no) invasion at 1h pi. Statistical analyses using a Mann Whitney test were performed. Relative values of significances were (****) p<0.0001; (***) p<0.0005; (**) p<0.01; (*) p<0.05.


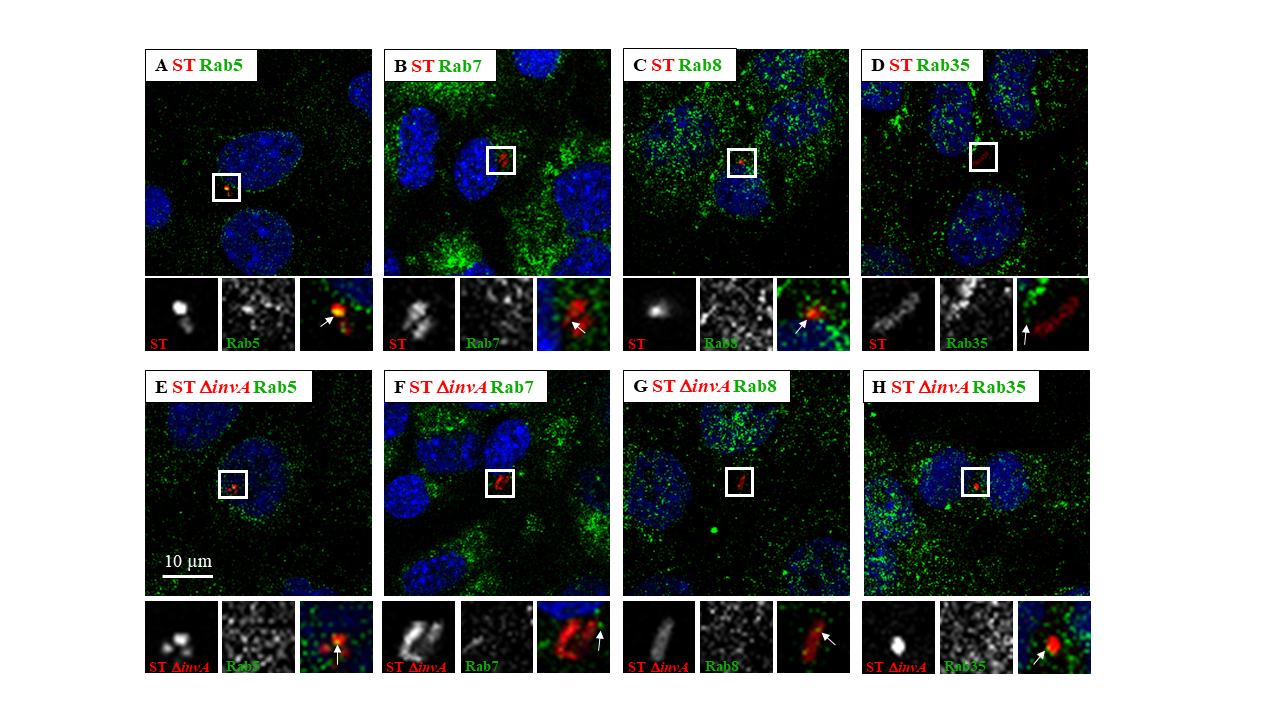


**Supplementary Figure** **3: *Salmonella* containing vacuole maturation occurred in weak mobilization of Rab GTPase.** AML12 cells infected with ST DsRed (A, B, C and D) and ST Δ*invA* DsRed (E, F, G and H) were fixed at 1 h pi, permeabilized and labelled with mouse Rab5 (A and E) or Rab7 (B and F) antibody or rabbit Rab8 (C and G) or Rab35 (D and H) antibody and revealed with anti-mouse or anti-rabbit Alexa Fluor 488 (green). Nuclei were counterstained with DAPI (blue). Squares outlined in white are magnifications of selected regions to visualize bacteria and Rab staining in black and white images beginning with a magnified merge image. The white arrow indicates the position of the Rab GTPase (green) near the bacteria (red). Scale bars 10 µm.

**
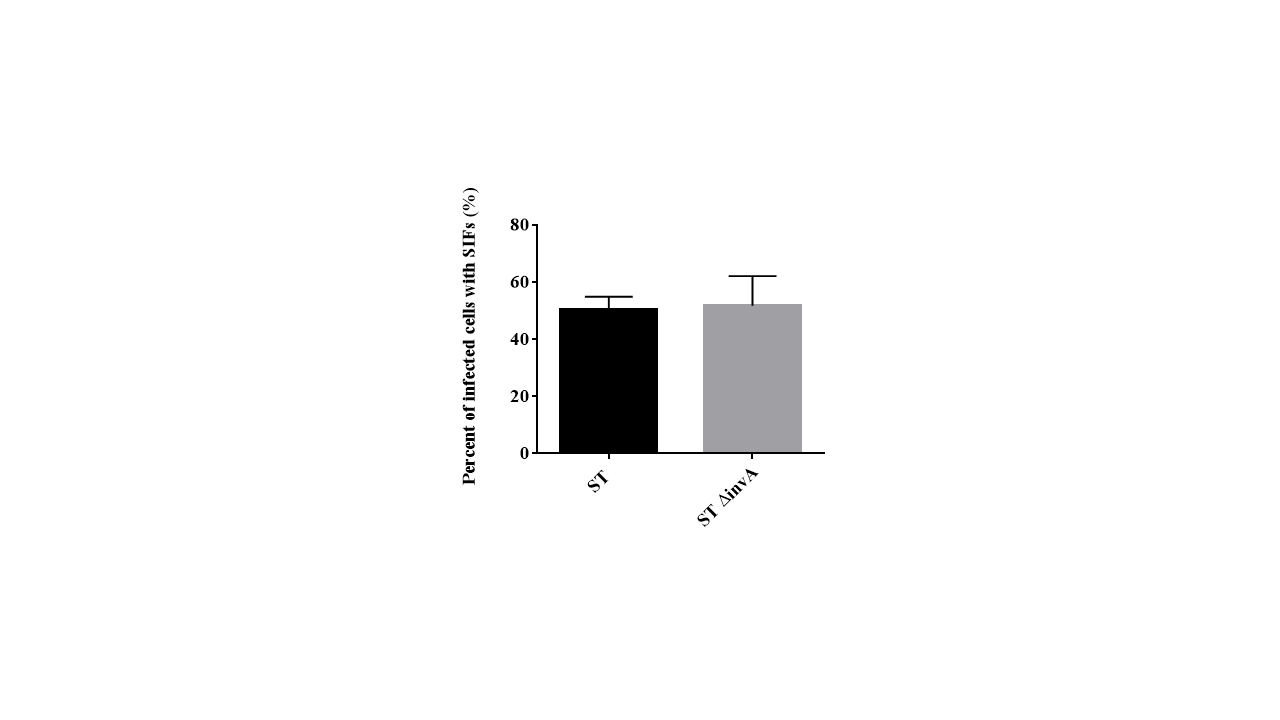
**

**Supplementary Figure 4: Half of the AML12 infected cells with *Salmonella* Typhimurium wild type or *Salmonella* Typhimurium *invA* mutant strains presented SIF.** AML12 cells infected with ST or ST Δ*invA* DsRed were fixed at 16h pi, permeabilized and labelled with rat Lamp1 antibody, and revealed using anti-rat Alexa Fluor 488. Over 100 *Salmonella* Typhimurium infected cells were observed by confocal microscopy; cells with SIF were counted. Data are the means and standard deviations from three independent triplicate experiments.


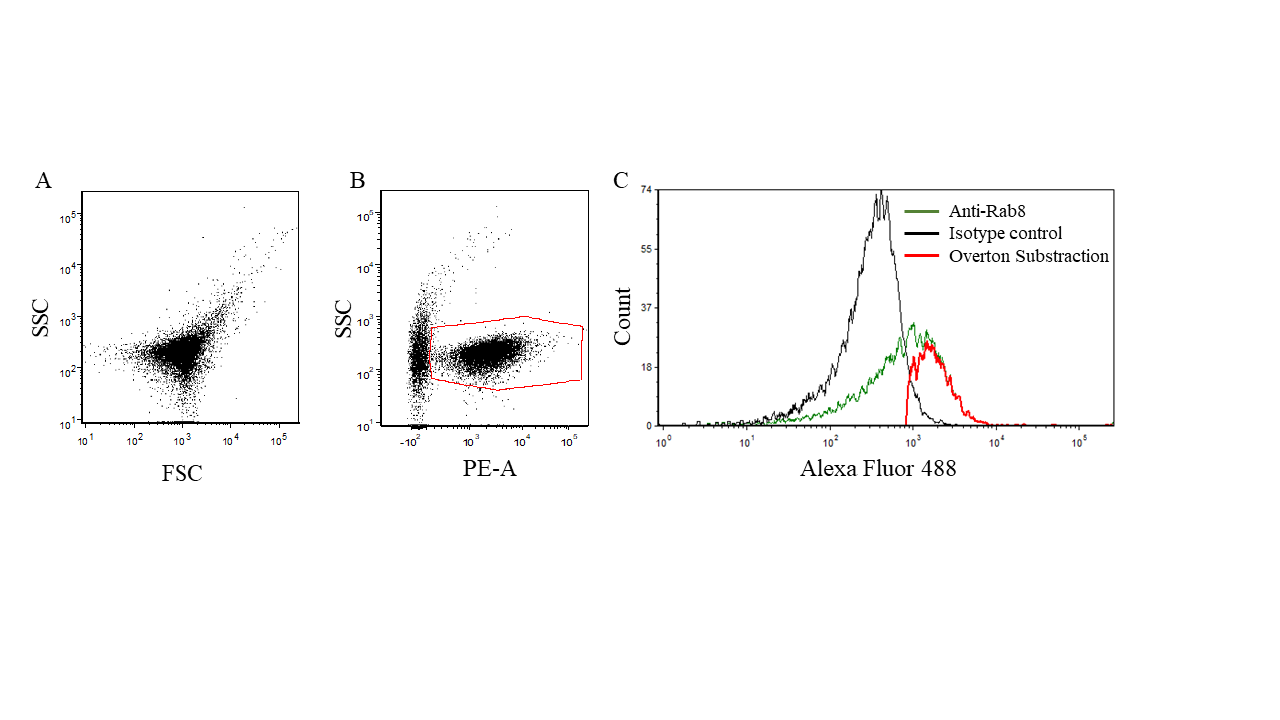


**Supplementary Figure 5: Analysis of Rab GTPase recruitment by flow cytometry.** PNS purified from AML12 cells infected 120 min with ST DsRed were labelled with Rab8 GTPase antibody and detected with Alexa Fluor 488 - conjugated goat anti–Rabbit (INVITROGEN). The relative fluorescence of the cell lines was analyzed using an LSR-Fortessa X-20 analyzer (BD BIOSCIENCES). (A) represents total PNS analyzed, (B) DsRed fluorescent PNS corresponding to SCV and (C) the relative recruitment of Rab8 on SCV expressed as the percentage of positive SCV determined by Overton subtraction of isotype control histograms from labelled histograms. Data represents10,000 events and are the means and standard deviations from three independent experiments.


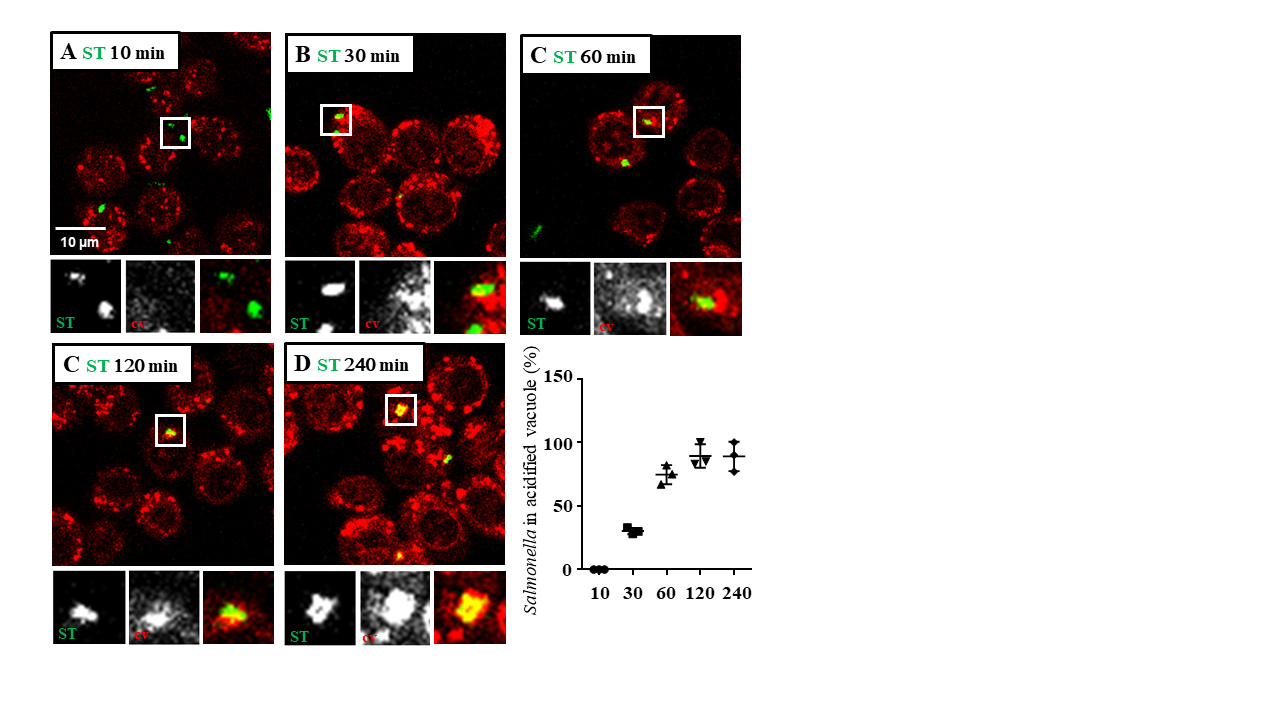


**Supplementary Figure 6: *Salmonella* Typhimurium progressed in acidified organelles in murine macrophages.** The kinetics of acidification was processed for RAW 264.7 cells infected with ST GFP at (A)10 min, (B) 30 min, (C) 60 min, (D)120 min and 240 min (E) pi, and SCV acidified was imaged and visually counted at each time point and (F) represented as percentage of SCV acidified over the total SCV. At each time point after 5 min exposure to 1 µM cresyl violet (cv) (red), cells were directly imaged by confocal microscopy with a 100x oil immersion objective (Leica TCS SP8, Germany). Squares outlined in white are a 2x magnification of selected regions in order to visualize progressive organelle acidification. Scale bars 10 µm.


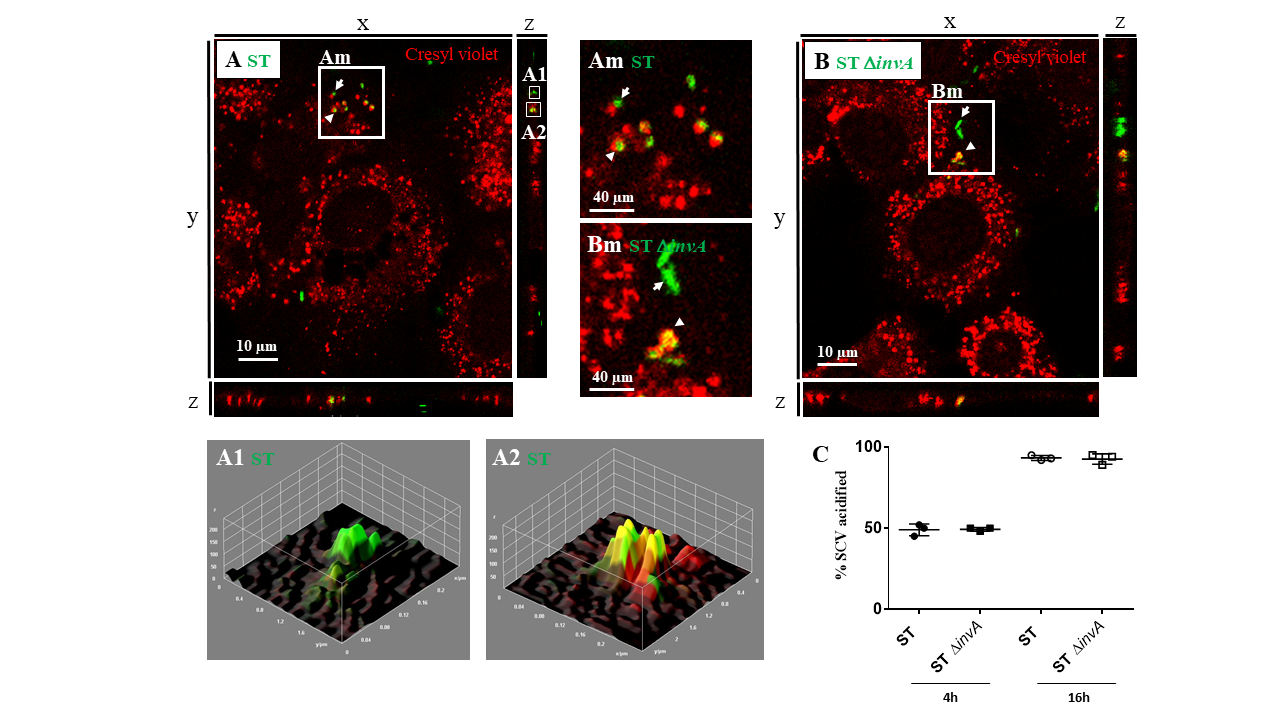


**Supplementary Figure 7: *Salmonella* Typhimurium 14028 resided in an acidified cell compartment.** At 4h pi, AML12 cells infected with (A) ST GFP or (B) ST Δ*invA* GFP were exposed for 5 min to 1 µM cresyl violet (red) and imaged in a z-stack setting using confocal microscopy with a 100x oil immersion objective (LEICA TCS SP8, Germany). Images of 1024x1024 pixels are presented in xy, xz and yz planes (A and B). Squares outlined in white represent a 2.5x manual magnification of selected regions to be able to visualize co-localization of bacteria with acidified cell organelles in an ST (Am) and ST Δ*invA* context (Bm). Selected regions of image A (A1 and A2) were processed in ImageJ software to construct 3D-colocalization plots (A1 and A2). Co-localization events were enumerated and are represented in bar graph (C). Error bars represent mean ± standard deviation of at least three independent experiments in which 100 bacteria (green) were enumerated for localization in acidified organelles (cresyl violet : red) for ST or ST Δ*invA*. Scale bars 10 µm, and 40 µm for the 2.5x manual magnification.
